# Sustainable ZnO-MIL-101(Fe)/Graphene-like Carbon Ternary Photocatalyst Composite (ZMG) for Efficient Sunlight-Driven Methyl Orange Degradation

**DOI:** 10.1021/acsomega.6c06086

**Published:** 2026-08-18

**Authors:** Armando Ramos Corona, Adriana Paola Franco Bacca, Raúl Pareja Rodríguez, José Ricardo Rangel Segura, Fernando Cervantes Alvarez, Rocio Martínez Flores, Jorge Rodríguez López, Geonel Rodríguez Gattorno, Juan José Alvarado Gil

**Affiliations:** † Cinvestav Unidad Mérida, Departamento de Física Aplicada, Carretera Antigua a Progreso Km. 6, Mérida, Yucatán 97310, México; ‡ Universidad Michoacana de San Nicolás de Hidalgo, Facultad de Ingeniería Química, Departamento de Posgrado, Gral. Francisco J. Múgica S/N, Ciudad Universitaria, Morelia, Michoacán 58030, México; § Instituto Tecnológico Superior de Monclova, Carretera 57, Km 4.5, Monclova, Coahuila 25733, México; ∥ Instituto Tecnológico del Valle de Morelia, Ciencias Básicas, Carretera Morelia Salamanca Km 6.5, Morelia, Michoacán 58100, México

## Abstract

Addressing the urgent need to convert biomass waste into high-value functional materials, this study presents the synthesis of a sustainable ternary photocatalyst: ZnO-MIL-101­(Fe)/Graphene-like carbon (ZMG). A central feature of this design is the strategic derivation of the graphene-like carbon (GLC) component from the abundant invasive biomass *Phragmites australis*, thereby aligning with Circular Economy principles. We evaluated the composite’s efficiency through the degradation of Methyl Orange (MO) under natural sunlight. Comparative evaluations demonstrated the superior performance of the ZMG system, achieving 85% MO removal under natural sunlight within 90 min, significantly surpassing the activity of both the individual components and binary composite. This enhanced activity is fundamentally due to an optimized synergistic relationship and an interfacial mechanism that ensures highly efficient charge-carrier separation. Under laboratory UV irradiation, the photocatalytic activity demonstrated excellent kinetics, achieving complete MO removal within 60 min and yielding the highest rate constant (κ = 0.070 min^–1^). Furthermore, the ZMG maintained excellent structural stability, with removal efficiency exceeding 96% after four consecutive reuse cycles. These findings validate the ZMG as a kinetically robust, sustainable, and highly effective candidate for practical wastewater remediation applications.

## Graphical abstract

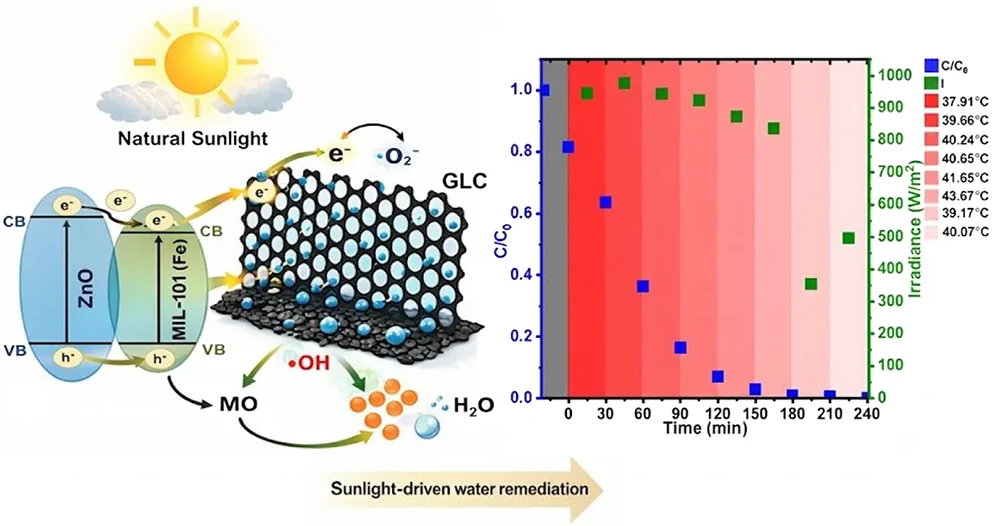

## 1 Introduction

The global demand for sustainable resource management has encouraged the development of strategies aligned with the principles of green chemistry and the circular economy. In this context, two correlated environmental challenges require urgent and innovative solutions: the contamination of water by organic dyes, a threat to aquatic ecosystems and public health,^1,2^ and the management of vast biomass waste quantities, representing a disposal problem and an underexploited source of materials to be harnessed.^3,4^ The use of residual biomass in advanced materials for water decontamination treatments exemplifies the principles of the circular economy, transforming environmental waste into a valuable resource.

Advanced oxidation processes (AOPs) are a promising set of technologies, with photocatalysis standing out as a technology that aligns with several key principles of green chemistry. The use of semiconductor catalysts, such as zinc oxide (ZnO), titanium dioxide (TiO_2_), cadmium sulfide (CdS), or tungsten trioxide (WO_3_), in combination with radiant energy eliminates the need for aggressive chemical reagents, thereby promoting energy efficiency. This approach generates highly reactive species capable of mineralizing organic pollutants into inoffensive byproducts such as CO_2_ and H_2_O.^5,6^

To further enhance sustainability and overall viability, current research focuses on developing highly efficient photocatalysts that operate under solar light, an abundant, renewable, and free energy source. In particular, zinc oxide (ZnO) is a wide-bandgap semiconductor (3.37 eV) with high chemical stability, low toxicity, low cost, and excellent photocatalytic activity toward a wide variety of organic contaminants.^7–10^ Owing to its high electron mobility and favorable band-edge positions, ZnO has been extensively investigated for dye degradation and environmental remediation under both UV and solar irradiation. However, the rapid recombination of photogenerated electron–hole (e^–^–h^+^) pairs and its limited visible-light absorption reduce its photocatalytic efficiency under solar irradiation. Recent studies have demonstrated that constructing ZnO-based heterostructures with carbon materials or other semiconductors effectively promotes interfacial charge separation, suppresses charge-carrier recombination, and enhances photocatalytic performance under visible or solar light.^11,12^ Consequently, the development of ZnO-based composite photocatalysts has become one of the most effective strategies to improve solar-light utilization and overall photocatalytic efficiency.^13^

A promising strategy to enhance photocatalytic performance is to develop composites integrating metal–organic frameworks (MOFs) with carbon-based materials. For example, MIL-101­(Fe) stands out as a candidate due to its highly porous structure and iron centers, which facilitate both contaminant adsorption and the generation of reactive oxygen species.^14,15^ Pairing graphene-like carbon (GLC) with MIL-101­(Fe) forms a system that leverages GLC’s excellent electrical conductivity, offering practical support for nanoparticle dispersion and enhancing charge transfer.^16,17^ As demonstrated in recent MIL-101-derived carbon composites, a high carbon content does not act as a mere passive baseline support, but rather as an essential 3D interconnected matrix that wraps and isolates individual microcrystals, preventing severe coalescence.^18^ A particularly sustainable approach includes synthesizing the carbon component from low-cost biomass waste, thereby converting an environmental liability into a functional, value-added material for photocatalysis.^19–21^ In this manner, an ecological challenge gets transformed into a high-value technological solution.

Recent advances in photocatalysis have increasingly focused on ternary heterostructures that combine semiconductors with conductive carbon materials to maximize interfacial charge transfer and suppress e^–^–h^+^ recombination. In particular, graphene-based matrices have proven highly effective for promoting charge separation because they provide conductive pathways for photogenerated electrons while simultaneously increasing the dispersion of the active components. Recent studies on ZnO-based and other ternary photocatalysts have demonstrated that this synergistic integration leads to improved photocatalytic efficiency and enhanced stability under light irradiation.^11,12,18,22^ Despite these advances, most reported graphene-based photocatalysts rely on graphene oxide synthesized from commercial graphite and are commonly evaluated under simulated laboratory illumination.

Recognizing the need for high-performance catalysts produced via sustainable synthetic routes, this work reports the preparation and characterization of a ternary photocatalyst, ZnO-MIL-101­(Fe)/GLC (ZMG), in which the GLC is derived from the invasive biomass *Phragmites australis*. This abundant, aggressively invasive macrophyte competes for water and displaces native biodiversity, yet it has no competing economic use and can be harvested year-round, so valorizing it as a carbon precursor turns an environmental liability into a functional material in line with Circular Economy principles. Its composition further supports this role, with a high volatile-matter content (∼70 wt %), an above-average fixed-carbon fraction (∼21 wt %), a low ash content (∼3 wt %), and an aromatic lignin fraction that promotes the formation of graphene-like domains upon thermal treatment. We evaluate the ZMG’s photocatalytic efficiency by measuring the degradation of methyl orange (MO) dye under both natural sunlight and UV irradiation. To establish a rigorous performance standard and demonstrate the synergistic effect, the individual components and the ZnO-MIL-101­(Fe) (ZM) binary composite were also studied. The degradation results confirmed the effective integration of all three components within the ternary architecture, resulting in significantly enhanced photocatalytic activity compared to its precursors, even under fluctuating solar irradiance. Moreover, the ZMG exhibited excellent stability and reusability over multiple cycles. These findings validate a circular-economy strategy that converts biological waste into a high-performance material, positioning ZMG as a durable, efficient photocatalyst for wastewater remediation.

## 2 Materials and Methods

### 2.1 Materials

The following analytical grade reagents as received without further purification were used in the synthesis of our materials: zinc acetate dihydrate ((Zn­(C_2_H_3_O_2_)_2_·2H_2_O), 99%, Alyt Chemicals), sodium hydroxide ((NaOH), 98%, JT Baker), terephthalic acid ((H_2_BDC, C_8_H_6_O_4_), 98%, Sigma-Aldrich), N,N-dimethylformamide ((DMF, C_3_H_7_NO), Sigma-Aldrich), iron­(III) chloride hexahydrate ((FeCl_3_·6H_2_O), 98%, Sigma-Aldrich), isopropyl alcohol ((C_3_H_8_O), Sigma-Aldrich), potassium hydroxide ((KOH), 99%, Sigma-Aldrich), and hydrochloric acid ((HCl), 37%, Sigma-Aldrich). Where needed, we used deionized water obtained from a LABCONCO WaterPro PS Polishing System.

### 2.2 Graphene-like Carbon (GLC) Synthesis

A biomass-derived graphene-like carbon was prepared from *Phragmites australis*. The biomass source was collected at the Monclova river region (26°57′01.1″N 101°24′26.8″W), located in Coahuila, northeast Mexico. First, the collected biomass was washed with tap water and distilled water to remove residual solid particles such as sand, clays and powder. The cleaned biomass was then sun-dried for 4 h to remove moisture and then cut into pieces of ∼1 × 3 cm to be ground with a commercial coffee grinder (Hamilton Beach, USA). Subsequently, the crushed biomass was sieved to get a particle size between 180 and 250 μm. Subsequently, 5 g of biomass was mixed with 70 mL of 1 M KOH solution and stirred at 300 rpm for 1 h. The solid material was then decanted and dried in an oven at 80 °C for 8 h. Subsequently, 5 g of the dried powder were thermally treated at 600 °C for 15 min in a muffle furnace (Thermo Scientific, USA) under atmospheric conditions, using a heating rate of 10 °C/min. To remove inorganic residues, the resulting carbonaceous material was dispersed in 50 mL of a 5% (v/v) HCl solution and stirred for 3 h. Finally, the product was washed with deionized water until the pH was neutral and then dried for subsequent use.

### 2.3 ZnO Synthesis

Zinc oxide (ZnO) was synthesized using a microwave-assisted hydrothermal method. In a typical procedure, 17.5 mL of a 0.5 M zinc acetate solution was dropwise added to 17.5 mL of a 2.0 M NaOH solution under constant magnetic stirring. The resulting mixture was stirred for an additional 30 min at room temperature and then transferred to a Teflon vial. The hydrothermal reaction was carried out in a microwave reactor (Anton Paar Multiwave 3000) by heating the vial to 200 °C at a rate of 10 °C/min and maintaining this temperature for 30 min, with a constant power of 600 W. After cooling to room temperature, the white precipitate was collected by filtration and washed several times with deionized water and isopropyl alcohol. The product was then dried in an oven at 90 °C for 24 h, followed by calcination in a muffle furnace at 400 °C for 4 h. Finally, the resulting powder was milled and sieved.

### 2.4 MOF-MIL-101­(Fe) Synthesis

The metal–organic framework MIL-101­(Fe) was synthesized via a microwave-assisted solvothermal method. A mixture of 0.4584 g of terephthalic acid (H_2_BDC) and 0.7460 g of iron­(III) chloride hexahydrate (FeCl_3_·6H_2_O) was dissolved in 35 mL of N,N-dimethylformamide (DMF) under magnetic stirring. The solvothermal reaction was then carried out in a microwave reactor by heating the solution at 200 °C for 50 min, with a heating rate of 5 °C/min and a constant power of 600 W. After cooling to room temperature, the resulting precipitate was collected and purified by suspending the solid in 30 mL of fresh DMF and stirring for 12 h to remove unreacted precursors. This washing step was repeated three times. Subsequently, the product was washed with isopropyl alcohol to remove the solvent, then dried in an oven at 120 °C for 12 h, yielding an orange powder.

### 2.5 Synthesis of the ZM (ZnO/MIL-101­(Fe)) Binary Composite

The ZM binary composite was synthesized via an in situ microwave-assisted hydrothermal method. The same volumes (8.75 mL) of 0.5 M zinc acetate and 2 M NaOH solutions were mixed and stirred for 30 min at room temperature to form a suspension. Then the suspension was poured into a Teflon vial containing 600 mg of the previously synthesized MIL-101­(Fe) powder. The vial with the reaction mixture was placed in a microwave reactor at 200 °C for 30 min, using a heating ramp of 10 °C/min and a power of 600 W. After the reactor cooled to room temperature, the resulting light-orange precipitate was collected by filtration. Finally, we washed the ZM composite repeatedly with deionized water and isopropyl alcohol and then dried it in an oven at 90 °C for 24 h.

### 2.6 Synthesis of the ZnO/MIL-101­(Fe)/GLC (ZMG) Ternary Composite

The ZMG ternary composite was synthesized using a simple ultrasonic dispersion method. The ZM composite powder and the as-prepared GLC powder were mixed in weight proportions of 30% and 70%, respectively, in 10 mL of isopropyl alcohol. This weight ratio was selected according to our previously reported methodology for GO-based semiconductor composites.^23^ The resulting suspension was sonicated for 2 h to ensure complete homogenization. Finally, to evaporate the solvent, the mixture was placed in an oven at 90 °C for 12 h, yielding the final ZMG ternary composite powder.

### 2.7 Characterization Techniques

We characterize the crystallinity of our samples by X-ray diffraction (XRD) analysis using a Bruker D8 Advance diffractometer in Bragg–Brentano mode, with Cu–Kα radiation (λ = 1.5418 Å), a step size of 0.02°, and a scan duration of 0.5 s. The morphology and elemental composition of the samples were examined using a field-emission scanning electron microscope (FE-SEM, JEOL JSM-7601F) equipped with an energy-dispersive X-ray (EDX) analyzer. To investigate the surface chemistry in greater detail, we conducted X-ray photoelectron spectroscopy (XPS) analysis using a Thermo Scientific K-Alpha instrument. Prior to analysis, the sample surface was briefly sputtered with Ar^+^ ions for 15 s to reduce the contribution of surface contaminants. We further explored the vibrational properties of the composites using Raman spectroscopy with a Witec 300 alpha spectrometer (633 nm excitation) and acquiring Fourier transform infrared (FTIR) spectra with an ATR attachment (Thermo Scientific Nicolet iS5), covering the range of 4000 to 500 cm^–1^. Finally, we evaluated the optical properties of the powders using an AvaSpec-2048 spectrophotometer with an integrating sphere and the surface area using the Brunauer–Emmett–Teller (BET) method.

### 2.8 Degradation Studies

To evaluate the photocatalytic activity, we prepared a suspension of 10 mg of photocatalyst in 40 mL of a 10 ppm MO solution. The catalyst loading (10 mg in 40 mL of a 10 ppm MO solution) was selected after preliminary experiments as a compromise between excessive adsorption and adequate photocatalytic evaluation and was kept constant for all samples to enable a direct comparison. The suspension was magnetically stirred in the dark for 30 min to ensure adsorption–desorption equilibrium. After this step, the photocatalytic degradation reaction was initiated by exposing the suspension to either natural sunlight or laboratory UV irradiation, depending on the experiment.

The sunlight experiments were conducted on a shade-free rooftop at Cinvestav Unidad Mérida (latitude 21.02°N, longitude 89.62°W). During each test, running from March to April 2025, we recorded solar irradiance and ambient temperature between 11:00 a.m. and 3:00 p.m. Aliquots of 1.75 mL were extracted every 30 min during this period of the day to determine the MO concentration via UV–vis spectroscopy (464 nm). For the laboratory UV irradiation tests, we used a 200 W lamp for 60 min. During this period, the MO suspension was tested at different intervals (every 5 min for the first 20 min, then every 10 min). The degradation percentage was calculated using the following equation:

1 $$D\;\left(\%\right)=\left(1-\frac{C}{C_{0}}\right)\times 100\%$$

where C_0_ is the initial dye concentration and C represents the MO concentration at different time intervals. The pseudo-first-order kinetic rate constant (κ) was determined by applying the Langmuir–Hinshelwood kinetic model, according to

2 $$\ln\frac{C_{0}}{C}=\kappa t$$

## 3 Results

### 3.1 Physicochemical Characterization

Figure 1 shows the diffractograms of each component and of the binary (ZM) and ternary (ZMG) composites. The ZnO diffractogram (Figure 1a) exhibits sharp, intense diffraction peaks corresponding to the hexagonal wurtzite phase (JCPDS No. 36-1451). The sharpness of these peaks indicates that the ZnO is highly crystalline, a desirable property for photocatalysis. The carbon obtained from *Phragmites australis* is a graphene-like carbon rather than graphene oxide. The XRD pattern (Figure 1b) shows the broad (002) band near 23.5° and the (100) in-plane reflection near 43.1°, characteristic of turbostratic graphene-like domains,^20,24,25^ confirming that the material is a graphene-like carbon and not an oxidized graphene phase. This behavior agrees with our previous work on the thermal treatment of this biomass.^21^ The diffractogram of MIL-101­(Fe) (Figure 1c) shows the characteristic peaks reported in the literature, confirming its successful synthesis and crystalline structure.^26,27^ The composites’ diffractograms confirm the successful integration of the components. On the one hand, the ZM binary composite diffractogram (Figure 1d) shows that the peaks of both the ZnO wurtzite phase and the MIL-101­(Fe) structure are clearly visible. This result indicates that in situ ZnO synthesis did not disrupt the MOF’s crystalline structure. On the other hand, the ZMG ternary composite diffractogram (Figure 1e) successfully combines the features of all three blended components. The sharp peaks from ZnO and the diffraction signals from MIL-101­(Fe) are preserved. Finally, the broad band corresponding to the (0 0 2) plane of GLC is also present. The coexistence of the three components’ diffractograms provides strong evidence for the successful formation of the ternary composite, a multiphase material designed to achieve potential synergistic effects.

**Figure 1:**
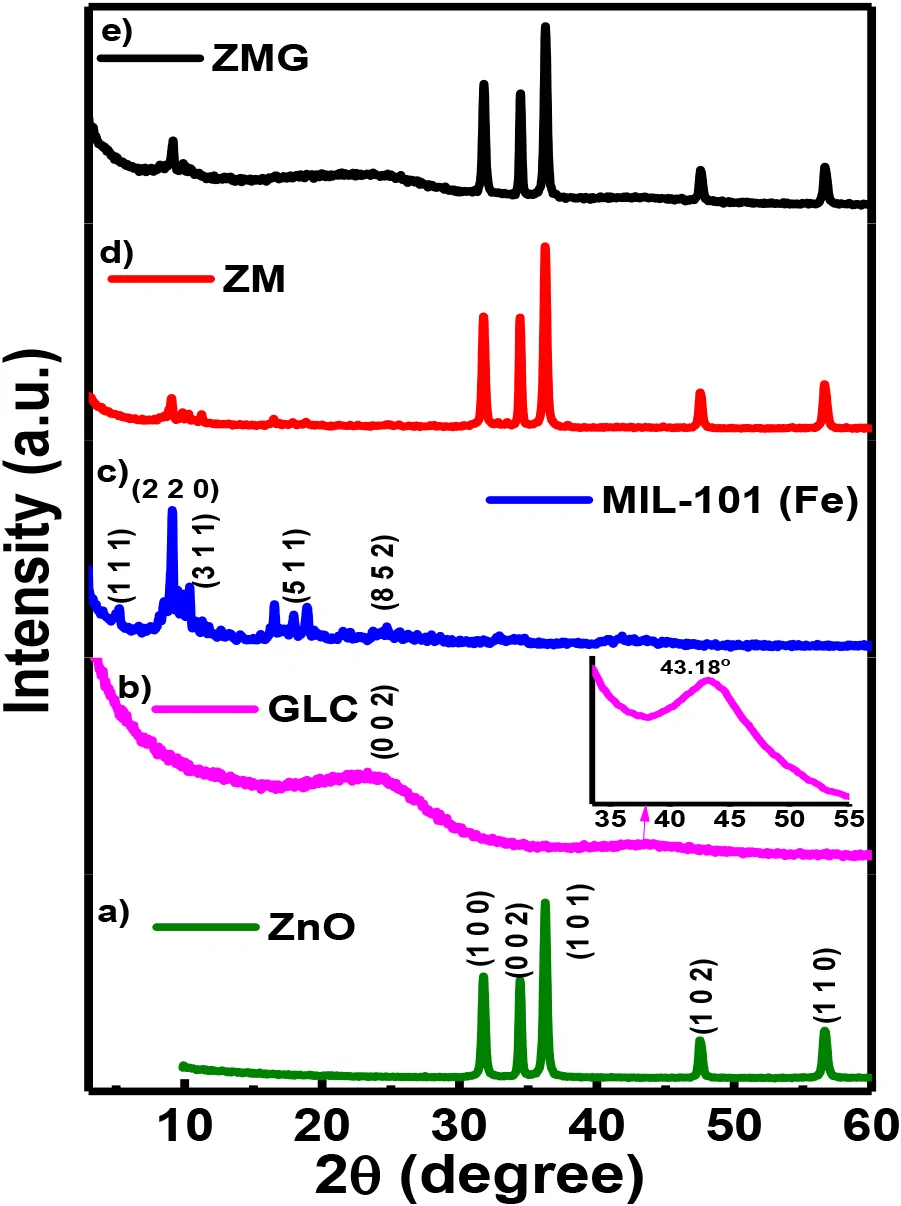
XRD patterns of a) ZnO, b) GLC, c) MIL-101­(Fe), d) ZM, and e) ZMG compounds.

Figure 2 presents SEM micrographs of synthesized materials, individual components, and composites. The ZnO sample (Figure 2a) exhibited a diverse morphology, consisting of nanostructures with both needle-like and rod-like shapes, as well as some hexagonal flakes. This structural variety is consistent with the crystalline features observed in the XRD diffractogram. The biomass-derived GLC (Figure 2b) exhibited a characteristic rough, porous surface with wide cavities, suggesting a large surface area suitable for integration with other materials, and enhancing charge transfer.^27^ As expected, the MIL-101­(Fe) sample micrograph (Figure 2c) shows well-defined octahedral crystals, a typical morphology for this iron-based MOF.^28^ In the ZM binary composite (Figure 2d), the micrograph shows less-defined particles that tend to form aggregates with ZnO nanoparticles assembled on and around the larger MIL-101­(Fe) crystals, indicating good contact between them. This close interaction is crucial, as it can promote a more efficient charge transfer during photocatalysis.^29,30^ Finally, the micrograph of the ZMG ternary composite (Figure 2e) reveals a highly rough and porous overall structure. The GLC resembles an interconnected carbonaceous scaffold, enabling the homogeneous dispersion of the ZM particles throughout its structure. This well-distributed and porous architecture is highly advantageous for photocatalysis, as it facilitates pollutant adsorption while promoting efficient charge separation and charge transport.^31^

**Figure 2:**
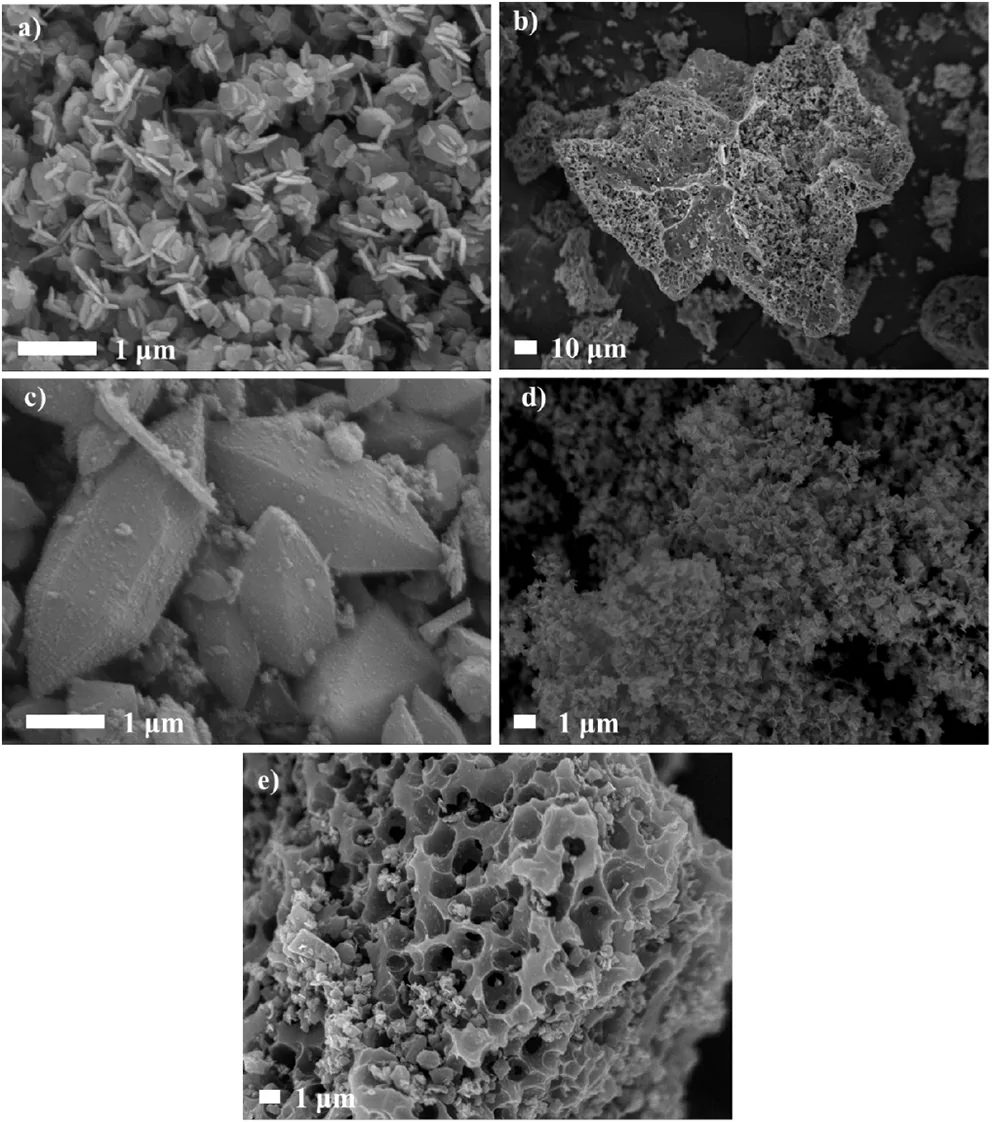
FE-SEM images of a) ZnO, b) GLC, c) MIL-101­(Fe), d) ZM, and e) ZMG.

Figure 3 shows the XPS survey spectra of the synthesized materials. The ZnO survey spectrum (Figure 3a) exhibits signals from the 2p states of Zn and the 1s states of O. The survey spectrum of GLC (Figure 3b) only presents the C 1s and O 1s signals. On the other hand, the MOF analysis (Figure 3c) reveals signals from its main elements: Fe 2p, O 1s, and C 1s. At the same time, the ZM binary composite spectrum (Figure 3d) clearly presents the signals of Zn 2p, Fe 2p, O 1s, and C 1s. Finally, the ZMG ternary photocatalyst composite exhibits the main Zn 2p, Fe 2p, O 1s, and C 1s signals, which confirms the coexistence of ZnO, the MIL-101­(Fe), and GLC on it.

**Figure 3:**
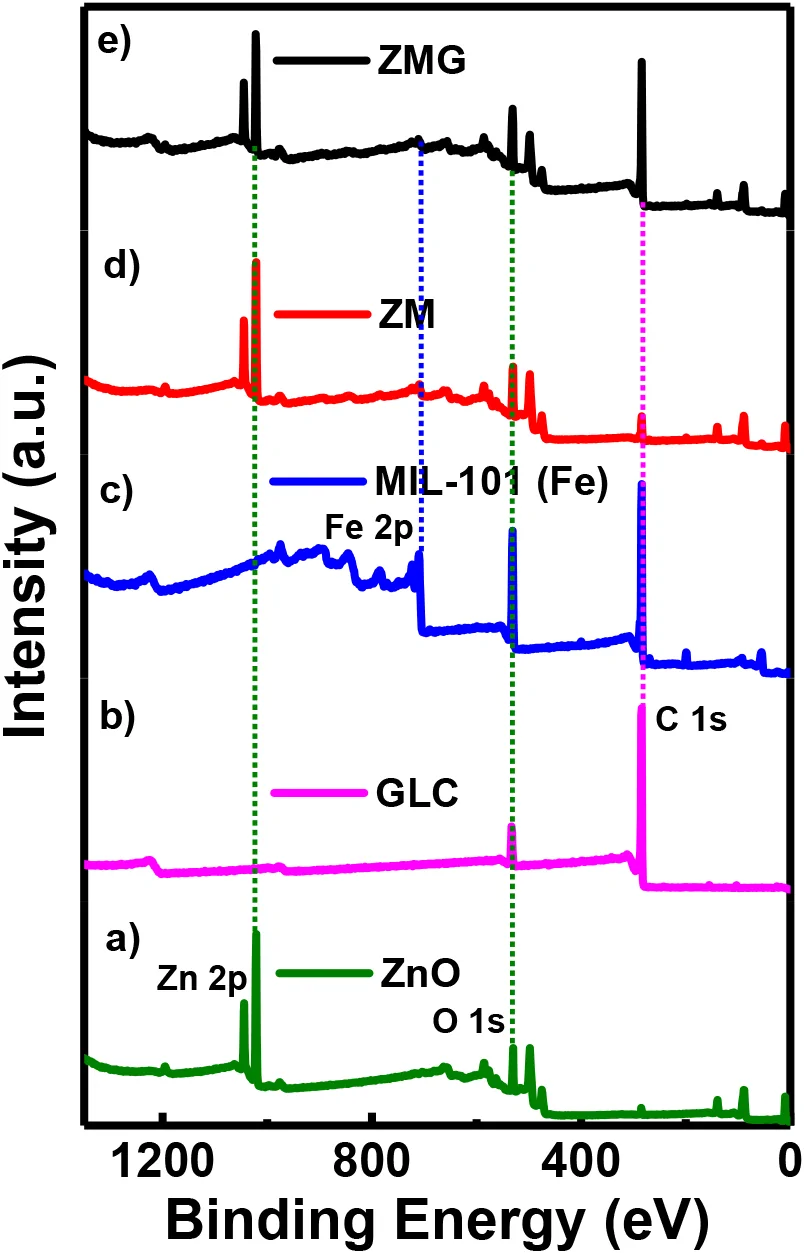
XPS survey spectra of a) ZnO, b) GLC, c) MIL-101­(Fe), d) ZM, and e) ZMG.

Figure 4a–c shows the HR spectra of the MIL-101­(Fe). After a deconvolution process, the HR spectrum for the Fe 2p state (Figure 4a) exhibits six contributions, which were identified as follows: two peaks at 710.43 eV (Fe 2p_3/2_) and 723.89 eV (Fe 2p_1/2_), indicating the presence of the Fe^2+^ oxidation state. The peaks at 715.34 eV (Fe 2p_3/2_) and 729.48 eV (Fe 2p_1/2_) indicate the presence of Fe^3+^ in the oxidation state. The peaks at 718.82 and 732.91 eV are satellite peaks of Fe^2+^ and Fe^3+^, respectively.^32^ The coexistence of both Fe^2+^ and Fe^3+^ species evidence a mixed iron oxidation environment, as a result of the synthesis conditions and the partial reduction of Fe^3+^ by DMF.^33^ Although a brief Ar^+^ sputtering treatment (15 s) was performed prior to XPS analysis to reduce the contribution of surface contaminants, the coexistence of Fe^2+^ and Fe^3+^ species has been consistently reported for MIL-101­(Fe), even in studies where no sputtering pretreatment was employed, suggesting that this mixed-valence state is an intrinsic characteristic of MIL-101­(Fe).^34–36^ The mixed-valence environment has been attributed to the coordination chemistry of Fe centers and the partial reduction of Fe^3+^ during solvothermal synthesis in DMF, and has been reported to promote Fe^2+^/Fe^3+^ redox cycling and more efficient charge transfer, thereby contributing to the enhanced photocatalytic performance of Fe-based MOFs.^33^ The HR spectrum of the O 1s state (Figure 4b) shows three main contributions at 530.20, 531.82, and 532.96 eV, corresponding to Fe–O bonds from the metal node of MIL-101­(Fe), C–O groups, and carbonyl C=O groups from the terephthalate ligand, respectively. On the other hand, the HR spectrum of the C 1s region (Figure 4c) reveals three peaks centered at approximately 284.73, 285.23, and 288 eV, attributed to C–C, C–O, and C=O bonds, respectively, which are consistent with the benzoic rings and the C=O of H_2_BDC.^35,37,38^

**Figure 4:**
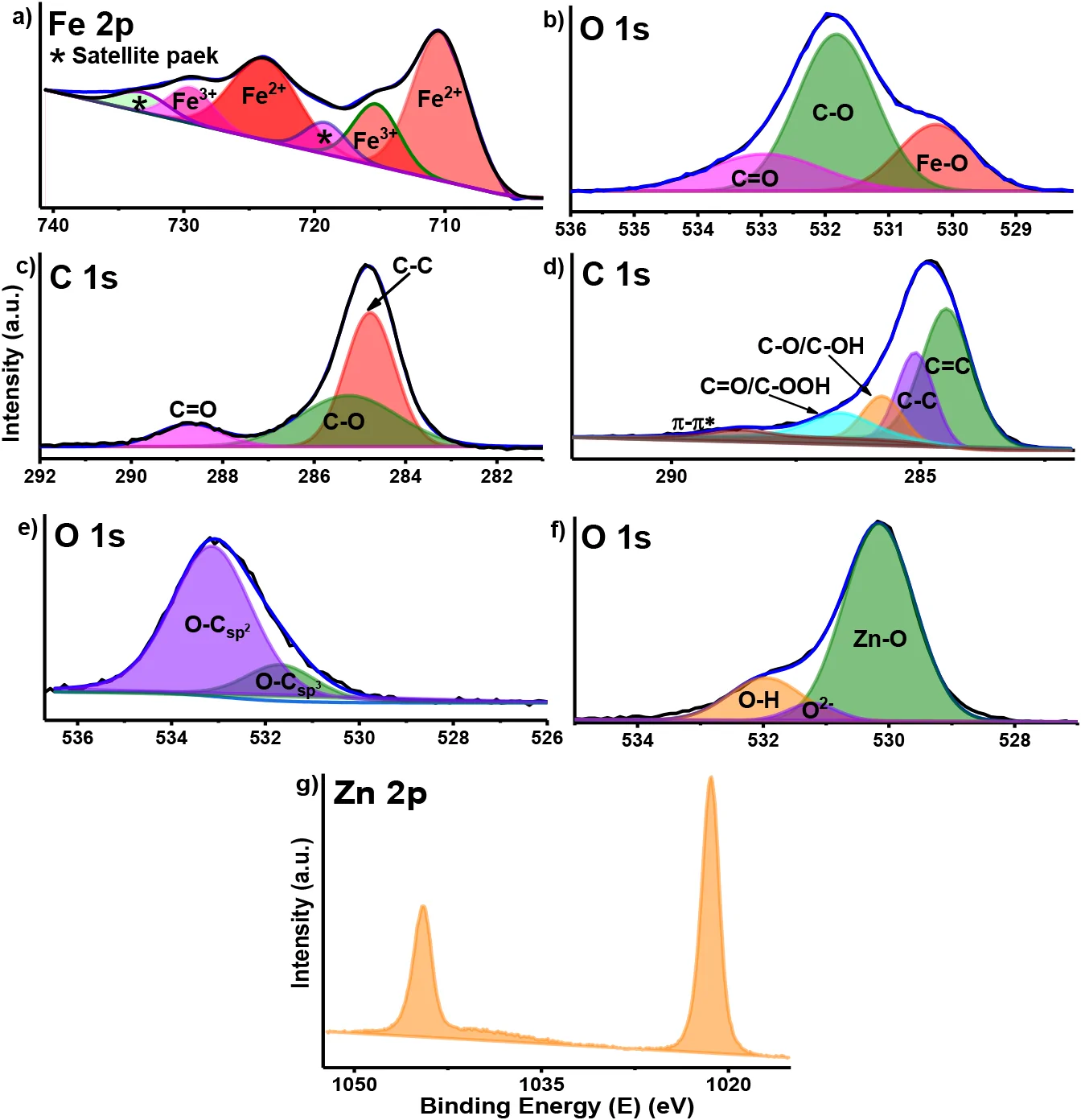
Deconvolutions of HR spectra for MIL-101­(Fe): a) Fe 2p, b) O 1s, and c) C 1s peaks; for GLC: d) C 1s, and e) O 1s peaks; and for ZnO: f) O 1s, and g) Zn 2p peaks.

The deconvolution of the HR spectrum corresponding to the C 1s peak of the GLC sample (Figure 4d) revealed several components attributable to different types of carbon bonding on the surface. Components associated with sp^2^-hybridized (C=C, 284.2 eV) and sp^3^-hybridized (C–C, 285.05 eV) carbon atoms related to the structural defects in the material’s 2D sheets were observed. Additionally, we identified oxygen-containing C–O and hydroxyl (C–OH) functional groups at 286.5 eV, and carbonyl and carboxylic groups (C=O, COOH) at 287.6 eV. The peak at 290.6 eV indicates the presence of aromatic carbons, characteristic of π–π* transitions, confirming the presence of carbons with two-dimensional character.^39^ Finally, the HR spectrum of the O 1s peak (Figure 4e) confirms the presence of O–Csp^3^ and O–Csp^2^ bonds, with peaks located at 531.25 and 533.4 eV, respectively.^40^

Figure 4f–g shows the HR spectra of the O 1s and Zn 2p regions of ZnO. The three main signals (Figure 4f) at 530.14, 531.21, and 531.97 eV correspond to Zn–O bonds, oxygen vacancies (O^2–^), and hydroxyl groups (−OH), respectively. It is reported that both −OH groups and O^2–^ stimulate the separation of e^–^–h^+^ pairs and thus improve the efficiency of photodegradation processes.^41^ In the Zn 2p region (Figure 4g), the two characteristic peaks located at 1021.38 and 1044.48 eV correspond to Zn 2p_3/2_ and Zn 2p_1/2_ signals, respectively. The peak positions indicate that zinc exists in the +2 oxidation state, which is typical for Zn^2+^ in the ZnO structure.^42^

Figure 5a–d presents the HR spectra of the ZM composite sample of the Fe 2p, C 1s, O 1s, and Zn 2p regions. The characteristic Fe 2p_3/2_ and Fe 2p_1/2_ peaks (Figure 5a) appear along with satellite signals in the Fe 2p region. Spectral deconvolution reveals the coexistence of Fe^2+^ and Fe^3+^ species, indicative of a mixed iron oxidation-state environment in the MOF. The C 1s spectrum (Figure 5b) shows three main components assigned to C–C (284.73 eV), C–O (285.23 eV), and C=O (288 eV) bonds, corresponding to the functional groups of the organic ligands. In the O 1s spectrum (Figure 5c), there are three well-defined contributions. The signal at 529.6 eV is attributed to Fe–O bonds from the MOF, while the one at 530.2 eV corresponds to Zn–O bonds characteristic of ZnO. The peak at 531.6 eV is associated with C–O groups from the organic ligands. Finally, in the Zn 2p region (Figure 5d), the Zn 2p_3/2_ and Zn 2p_1/2_ peaks at 1021.4 and 1044.5 eV confirm the presence of Zn^2+^. The analytical suite confirms the coexistence and integration of ZnO and MIL-101­(Fe) phases in the ZM composite.

**Figure 5:**
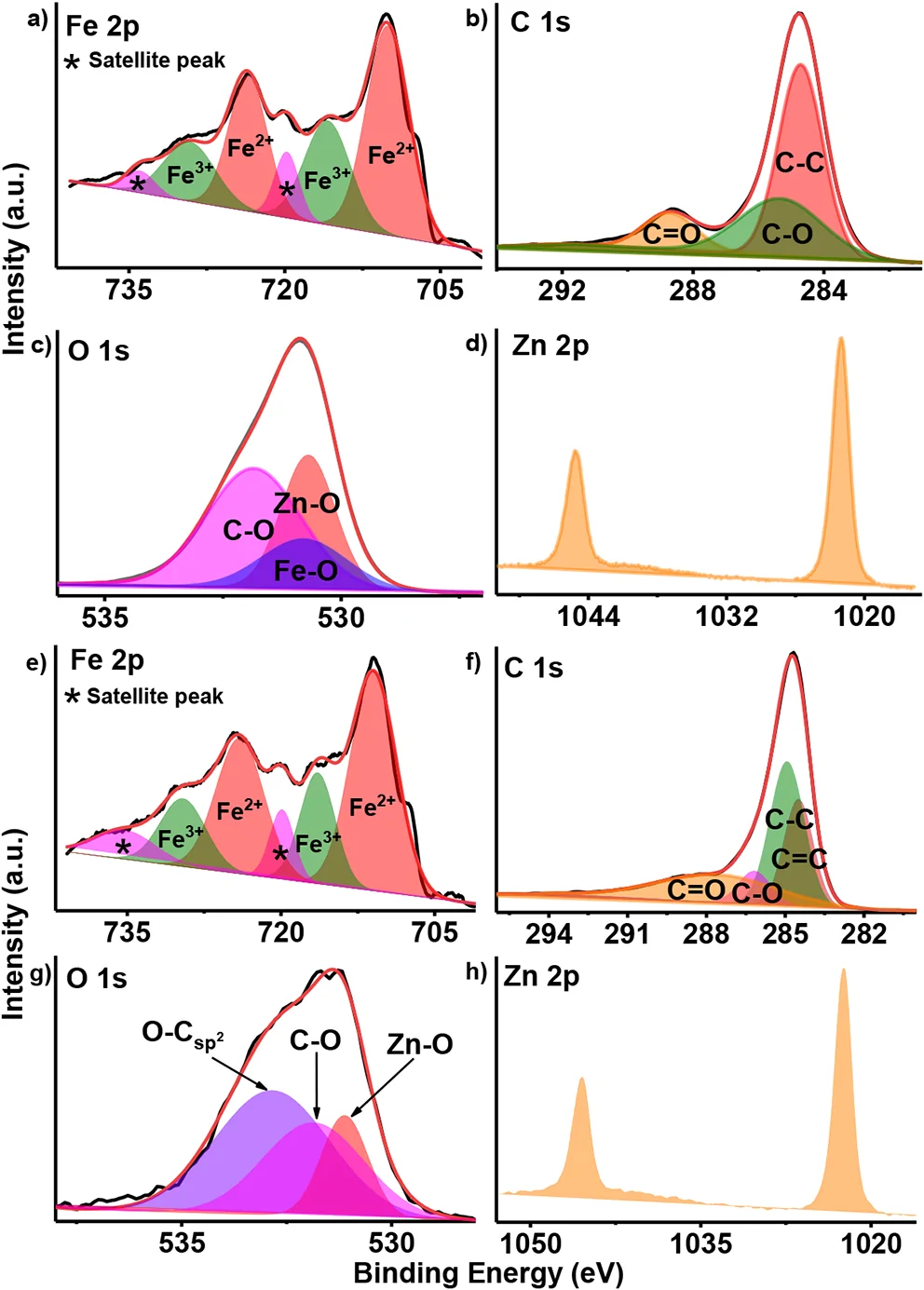
HR spectra of the ZM and ZMG compounds. For ZM: a) Fe 2p peak, b) O 1s peak, c) C 1s peak, and d) Zn 2p peak. For ZMG: e) Fe 2p peak, f) C 1s peak, g) O 1s peak, and h) Zn 2p peak.

Figure 5e–h shows the XPS HR spectra of the ternary ZMG composite. In the Fe 2p region (Figure 5e), the characteristic peaks of Fe^2+^ and Fe^3+^ are observed, along with satellite signals, indicating a mixed iron oxidation environment derived from the MOF. The C 1s spectrum (Figure 5f) presents four components: C=C (284.49 eV), C–C (284.91 eV), C–O (286.20 eV), and C=O (287.82 eV), which correspond to the functionalities present in both the MOF organic ligands and GLC particles. In the O 1s window (Figure 5g), three contributions are observed at 531 eV (Zn–O), 531.73 eV (C–O), and 532.83 eV (O–Csp^2^), corresponding to both the ZnO inorganic network and the oxygenated groups of MOFs and GLC. Finally, in the Zn 2p region (Figure 5h), the Zn 2p_3/2_ and Zn 2p_1/2_ peaks at 1021.5 and 1044.6 eV, respectively, confirm the Zn^2+^ oxidation state. These results support the coexistence and interaction of the three components in the ZMG composite.

Figure 6a presents the DRS spectra of the samples, with ZnO exhibiting a maximum at 460 nm and a reflectance of approximately 90%. MIL-101­(Fe) and ZM have similar spectra. However, ZM shows higher reflectance between 350 and 570 nm than the MOF, likely due to the incorporation of ZnO. From 350 nm onward, the reflectance percentage is lower than that of MIL-101­(Fe), reaching a maximum of 57% at 740 nm. MIL-101­(Fe) reaches a maximum of 64% at 740 nm. These results indicate that both materials absorb visible radiation. Using the Kubelka–Munk (K-M) function (see eq 3), we estimate the optical bandgap.

3 $$F\left(R\right)=\frac{{\left(1-R\right)}^{2}}{2R}=\frac{K}{s}\approx \alpha =\frac{{\left(h\upsilon -E_{g}\right)}^{n/2}}{h\upsilon }$$

where *R* is the diffuse reflectance, *K* is the absorption coefficient, *s* is the scattering coefficient, *hν* is the photon energy, E_g_ is the bandgap energy, and *n* can take the values 1 or 4 for indirect and direct bandgap semiconductors, respectively.^43,44^ For ZnO, which behaves as a direct semiconductor, the optical E_g_ is obtained by extrapolating the tangent to the (αhν)^2^ vs hν plot (Figure 6b), yielding an E_g_ of 3.2 eV. In contrast, for MIL-101­(Fe) and the ZM composite, this method is not applicable because of their optical absorption from complex ligand-to-metal charge transfer (LMCT), π–π*, and spin-forbidden d-d transitions; therefore, only the shift in their optical absorption edge is shown in Figure 6c–d without assigning a bandgap value.

**Figure 6:**
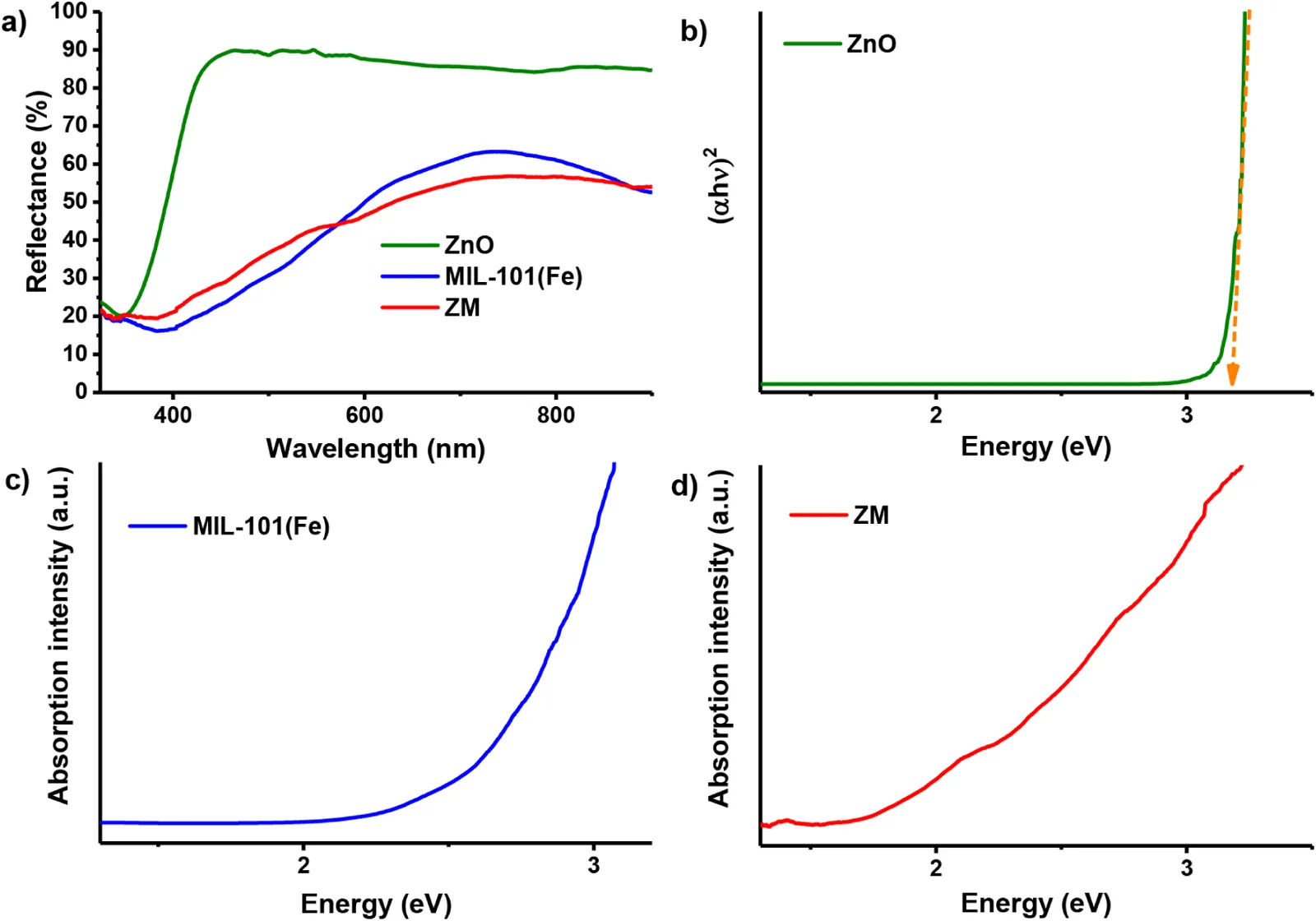
a) diffuse reflectance spectra of ZnO, MIL-101­(Fe), and the ZM, b) optical bandgap of ZnO, c) optical absorption edge of MIL-101­(Fe), and d) optical absorption edge of the ZM.

Light absorption plays a pivotal role in the photocatalytic efficiency of the ZMG composite. Figure 6 shows UV–vis electronic absorption spectra of the systems under study. ZnO primarily absorbs UV light, while MIL-101­(Fe) extends the absorption into the visible region, enabling better utilization of the solar spectrum. The incorporation of GLC further enhances photon harvesting by improving light scattering and facilitating charge transport. This synergistic absorption profile ensures that ZMG captures a broader range of sunlight, generating more electron–hole pairs and accelerating pollutant degradation under real solar conditions. Note that we will use the term “bandgap” only for ZnO, while for the other materials we will refer solely to their optical edge. Systems such as MOFs are known for having strong absorption bands in the UV region (below 400 nm), primarily due to LMCT and interlinker π–π* transitions. All possible d-d electronic transitions from this sextet ground state to excited quartet states (such as^4^A_1g_, ^4^E_g_, ^4^T_1g_, ^4^T_2g_) violate the spin selection rule (ΔS = 0). This condition makes the transitions parity-forbidden, resulting in very low intensity in the UV–vis spectrum. Any attempt to analyze the presence of a bandgap using the “Tauc plot” could be an overinterpretation because most probably the nature of the transitions is complex (direct and indirect mixing) and/or overlapped transitions between nonparabolic bands.^45^

The Raman ZnO spectrum (Figure 7a) exhibits a first peak at 99 cm^–1^, which is the E_2_ (low) mode, corresponding to the vibration of zinc (Zn) within the crystalline structure of ZnO.^46^ The second peak at 438 cm^–1^ is the E_2_ (high) mode, arising from the vibration of the oxygen (O) atom, indicating vibrational coupling between the O and Zn atoms. Weaker signals at 337 cm^–1^, 583 cm^–1^, and 1155 cm^–1^, correspond to the E_2_ (high) – E_2_ (low), E_1_ (LO), and 2E_1_ (LO) modes, respectively.^47^ These vibrations are characteristic of the wurtzite structure of ZnO.

**Figure 7:**
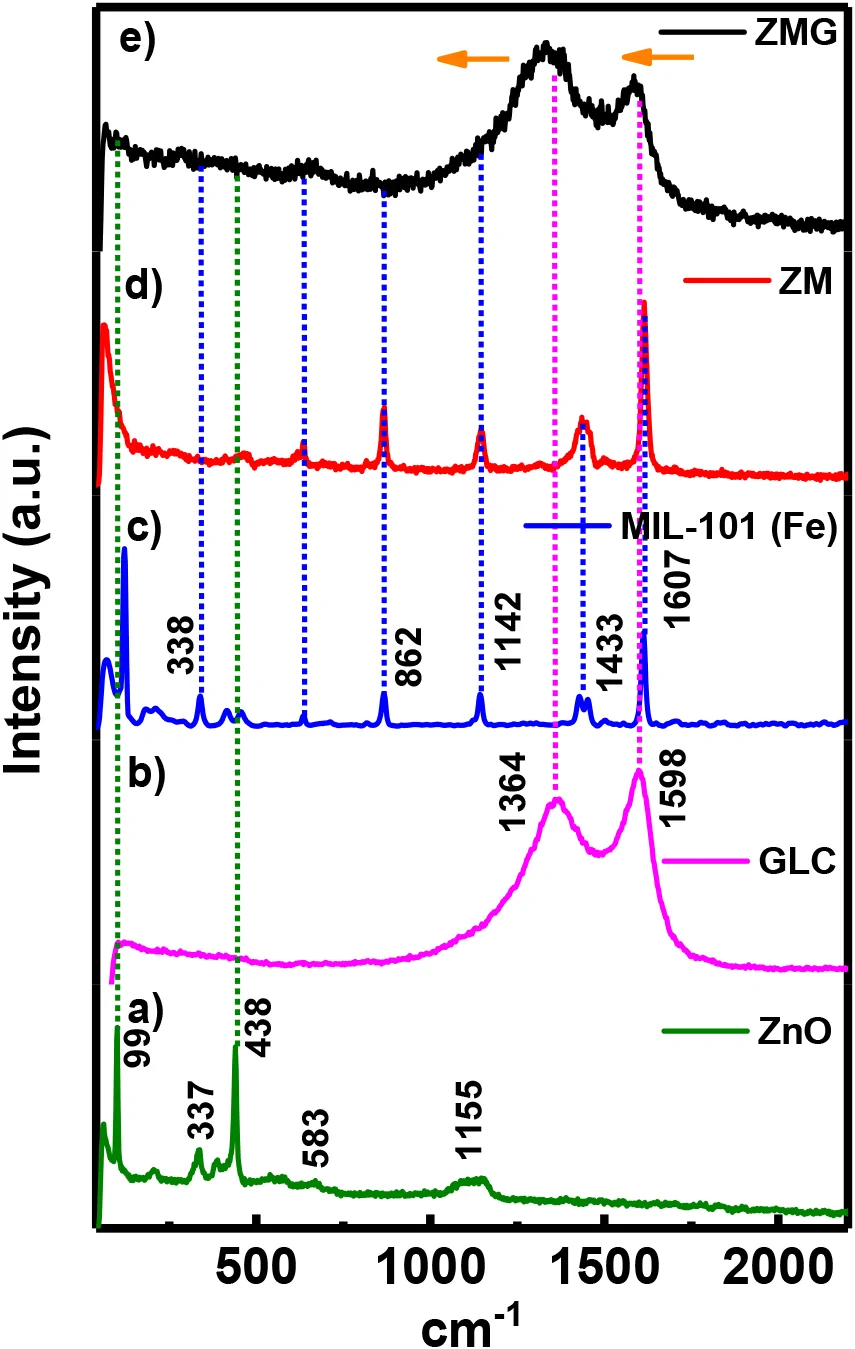
Raman spectra of a) ZnO, b) GLC, c) MIL-101­(Fe), d) ZM, and e) ZMG.

The Raman spectrum of the GLC (Figure 7b) displays the two bands characteristic of disordered graphene-like carbons: a well-defined G band at 1598 cm^–1^ and a D band at 1364 cm^–1^. The G band arises from the in-plane stretching of sp^2^ (C=C) pairs and confirms the presence of ordered aromatic domains; its prominence agrees with the dominant sp^2^ C=C component resolved in the C 1s XPS spectrum (284.2 eV, Figure 4d) and with the turbostratic graphene-like stacking denoted by the broad (002) and (100) reflections in the XRD pattern (see Figure 1b).^48,49^ The D band, in turn, reflects structural disorder together with edge and boundary defects of the sp^2^ domains, consistent with a nanostructured, turbostratic carbon rather than with extensive basal-plane oxidation.^50,51^ This interpretation is supported by XPS, where the oxygen-bearing groups (C–O/C–OH at 286.5 eV and C=O/COOH at 287.6 eV, with weak O 1s signals) constitute only a minor fraction confined to the sheet edges; such limited functionalization contrasts sharply with graphene oxide, whose heavy basal-plane oxidation produces a markedly stronger D band, a broadened and upshifted G band, and the (001) reflection near 10° that is absent in our material. Together with the π–π* shakeup satellite observed by XPS (290.6 eV), the coexistence of a strong G band and a moderate D band confirms that the sample is a graphene-like carbon with an extended sp^2^ network and only peripheral oxygenation.

The Raman spectrum of MIL-101­(Fe) (Figure 7c) shows several relevant bands, particularly associated with the organic ligands present in its structure. Vibrations of the benzene ring and the C–H bond correspond to the band at 862 cm^–1^, suggesting the presence of aromatic groups in the structure. These vibrations are crucial for understanding the material’s stability and functionality.^26^ The bands at 1145 cm^–1^ and 1433 cm^–1^ are from the C–C bond of the benzene ring and the C=O bond in the carboxylic group, respectively. Finally, the band at 1613 cm^–1^ corresponds to the antisymmetric stretching vibration of the carboxylate group (−COO^–^). All these bands indicate the structural integrity of the organic ligands, which are fundamental to MIL-101­(Fe)’s properties.

The bands in the Raman spectrum of the ZM binary composite (Figure 7d) indicate the predominance of the MOF structure in the analyzed sample. They result from the specific vibrations of the organic ligands and the MOF’s metallic structure. However, some modes associated with ZnO are also identified, albeit at lower intensity. This suggests that, despite the integration of ZnO into the MOF matrix, the contribution of the ZnO vibrations is less significant compared to those of the MOF. Their presence indicates that ZnO is effectively incorporated into the composite, thereby influencing the composite’s functional properties.

The Raman spectrum of the ZMG ternary composite (Figure 7e) shows signals from MIL-101­(Fe) overlapping in the 900–1850 cm^–1^ range, with the dominant G and D bands of GLC. Additionally, blue shifts are observed in specific spectral bands. This phenomenon implies significant interactions among GLC, ZnO, and MIL-101­(Fe), suggesting structural coupling and possible modifications to the electronic and vibrational properties of the individual components. Blue shifts often indicate an increase in vibrational energy resulting from internal stresses or strong interactions within the composite system.

The FTIR spectrum of ZnO (Figure 8a) shows two significant vibrational bands at 520 and 677 cm^–1^, assigned to the stretching modes of the Zn–O bond.^52^ The small bands in the C–H region, specifically at 880 cm^–1^, correspond to C–H stretching vibrations, suggesting the presence of organic contaminants on the ZnO surface. The band at 3400 cm^–1^ corresponds to O–H stretching vibrations, indicating the adsorption of hydroxyl groups on the material’s surface.^52^ Finally, the band at 2360 cm^–1^ arises from adsorbed CO_2_, either from the sample itself or from the environment, a feature common in studies of materials exposed to environmental conditions.^53^

**Figure 8:**
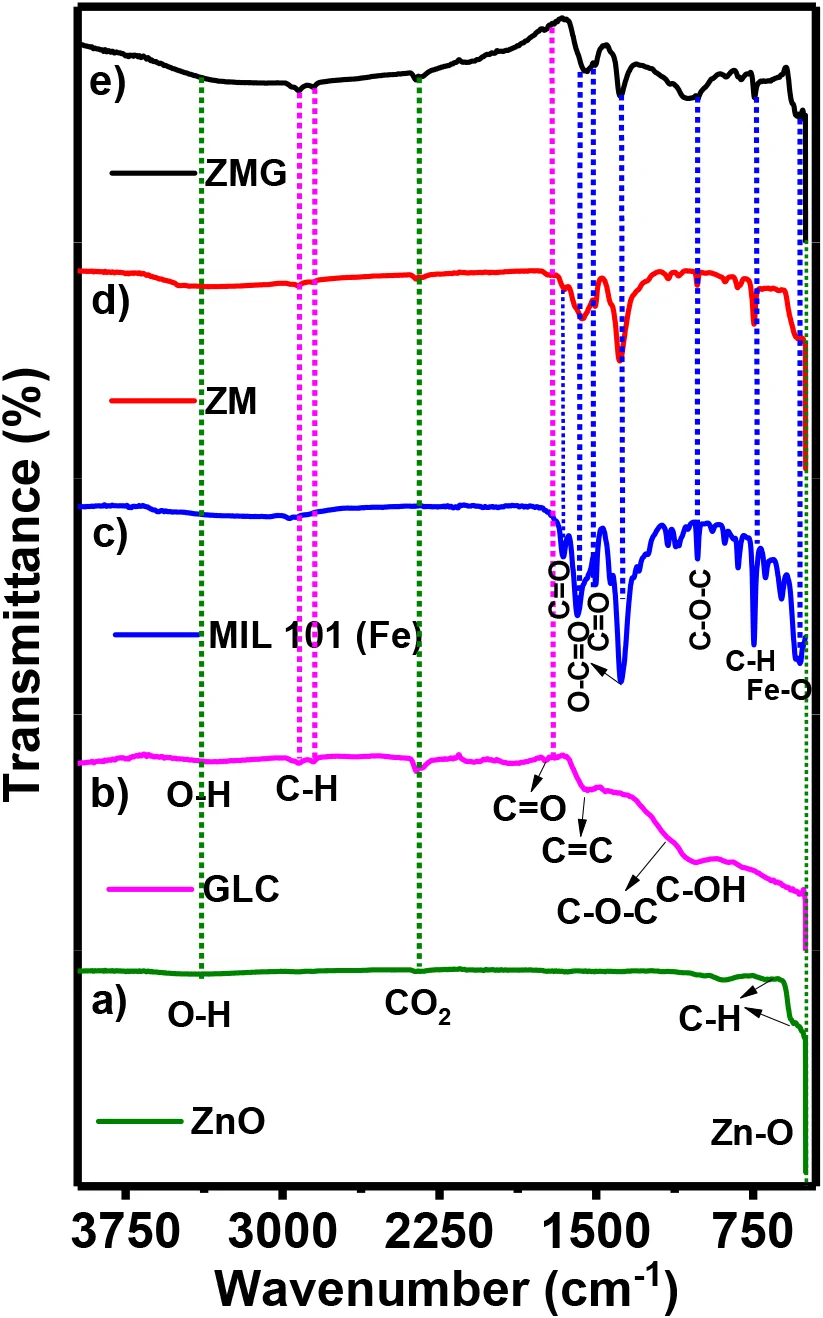
FTIR spectra of a) ZnO, b) GLC, c) MIL-101­(Fe), d) ZM, and e) ZMG.

The GLC’s FTIR spectrum (Figure 8b) presents a series of significant vibrational bands. The first at 1036 cm^–1^ corresponds to the vibration of hydroxyl groups (C–OH), suggesting the presence of surface functionality on the GLC.^50^ Additionally, the band at 1170 cm^–1^ is associated with C–O–C groups, indicating that the GLC structure has bonds that contribute to its stability and chemical properties.^50^ The stretching vibration of the carbon–carbon double bond (C=C) appears at 1560 cm^–1^,^20,50^ and at 1745 cm^–1^, the band of carbonyl groups (C=O) located at the edges of the GLC appears.^54^ This band suggests surface functionalization and may affect the material’s adsorption properties. Bands from the C–H bonds at 2840 cm^–1^ and 2920 cm^–1^ result from symmetrical and asymmetrical stretching vibrations, respectively.^54,55^ These bands indicate the number of methyl and methylene groups in the GLC. Finally, the stretching vibration of hydroxyl groups (O–H) at 3400 cm^–1^ indicates the presence of adsorbed water or functional groups that may contribute to the chemical reactivity of GLC.^54^

The MIL-101­(Fe) FTIR spectrum (Figure 8c) exhibits a peak at 540 cm^–1^ related to the vibration of the Fe–O bond.^26,27^ Bending vibration out of the plane of the C–H bonds of the benzene ring of H_2_BDC appears at 747 cm^–1^. The bands at 1500 cm^–1^ and 1670 cm^–1^ arise from the asymmetric stretching of the carboxyl groups linked by C=O in H_2_BDC. Additionally, C–O–C vibrations appear at 1017 cm^–1^, and the bands at 1384 cm^–1^ and 1580 cm^–1^ are assigned to the symmetric and asymmetric O–C=C vibrations, respectively. The bending modes for the COO groups at 630 cm^–1^ correspond to unreacted terephthalic acid.^29^ Finally, the broad band around 3400 cm^–1^ results from the stretching vibration of water molecules (H_2_O).

The FTIR spectra of the ZM (Figure 8d) and ZMG (Figure 8e) composites show no significant differences or new vibrations compared to those of ZnO, MIL-101­(Fe), and GLC. However, there is some overlap between the bands, indicating that although the spectral characteristics of each component are maintained, the combination of ZnO, MIL-101­(Fe), and GLC influences the vibrational interactions among the materials.

Using the Brunauer–Emmett–Teller (BET) method, we determined the surface area through physical N_2_ adsorption and desorption as a function of relative pressure (P/P_0_). Figure 9 shows the surface area results, revealing that ZnO has a surface area of 15.86 m^2^/g, consistent with values reported in the literature using various synthesis methods.^29,56,57^ The MIL-101­(Fe) exhibited a surface area of 384.33 m^2^/g, a value higher than that reported for other materials synthesized by solvothermal methods.^43^ GLC displayed the highest surface area, reaching 530.55 m^2^/g, this high specific surface area, the largest among the individual components, reflects the well-developed, exfoliated sheet-like texture of the graphene-like carbon and provides an extensive scaffold that promotes the effective dispersion of ZnO and MIL-101­(Fe), maximizing interfacial contact and the availability of active sites for charge transfer and pollutant adsorption. The ZM binary composite, compared to its components, exhibited a significant decrease relative to MIL-101­(Fe), achieving a BET surface area of 177.02 m^2^/g. In this case, ZnO particles likely partially fill the MOF structure.^43,58^ The average pore diameters for ZnO, MIL-101­(Fe), ZM, and GLC are 23.19, 1.42, 1.75, and 2.88 nm, respectively. On the ZM particles supported on GLC, it was observed that the high surface area of GLC was largely preserved, with only a slight reduction up to 484.41 m^2^/g and a pore size diameter of 2.74 nm. This result suggests that GLC acts as an efficient support, allowing the ZM particles to remain well distributed without significantly altering GLC’s textural properties, which remain almost intact.

**Figure 9:**
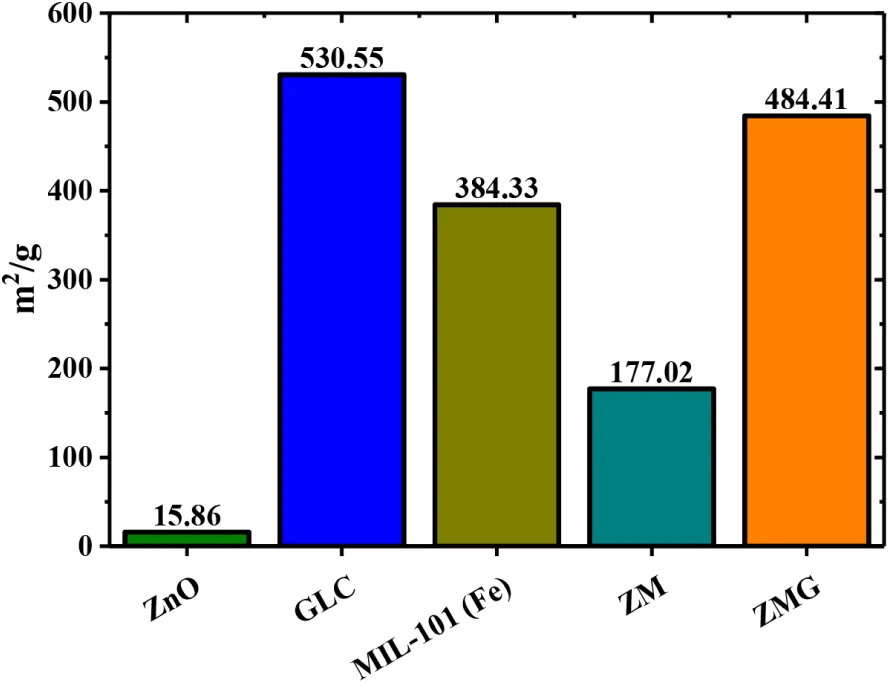
Surface area values for the synthesized compounds.

### 3.2 Photocatalytic Tests

The synthesized components and composites were evaluated as photocatalysts for the degradation of MO under natural sunlight, which was monitored by UV–Vis spectroscopy. Figure 10 presents the photocatalytic degradation performance of the synthesized materials under sunlight irradiation. To evaluate photolysis-induced side effects from the dye under solar exposure, we conducted a control experiment that excluded the photocatalyst. No significant change in MO concentration was observed during the control photolysis experiment, as shown in Figure 10a, confirming that the decrease in the characteristic MO absorption band observed in the subsequent experiments is attributable to the combined action of the photocatalyst and solar irradiation. All suspensions were stirred in the dark for 30 min to achieve adsorption–desorption equilibrium. This period is represented by the gray bar at the beginning of all plots. The reaction was initiated immediately after, by placing the solution under solar irradiation, taking this moment as time zero. During the degradation tests, both irradiance and ambient temperature were monitored and recorded as average values for each 30 min sampling interval.

**Figure 10:**
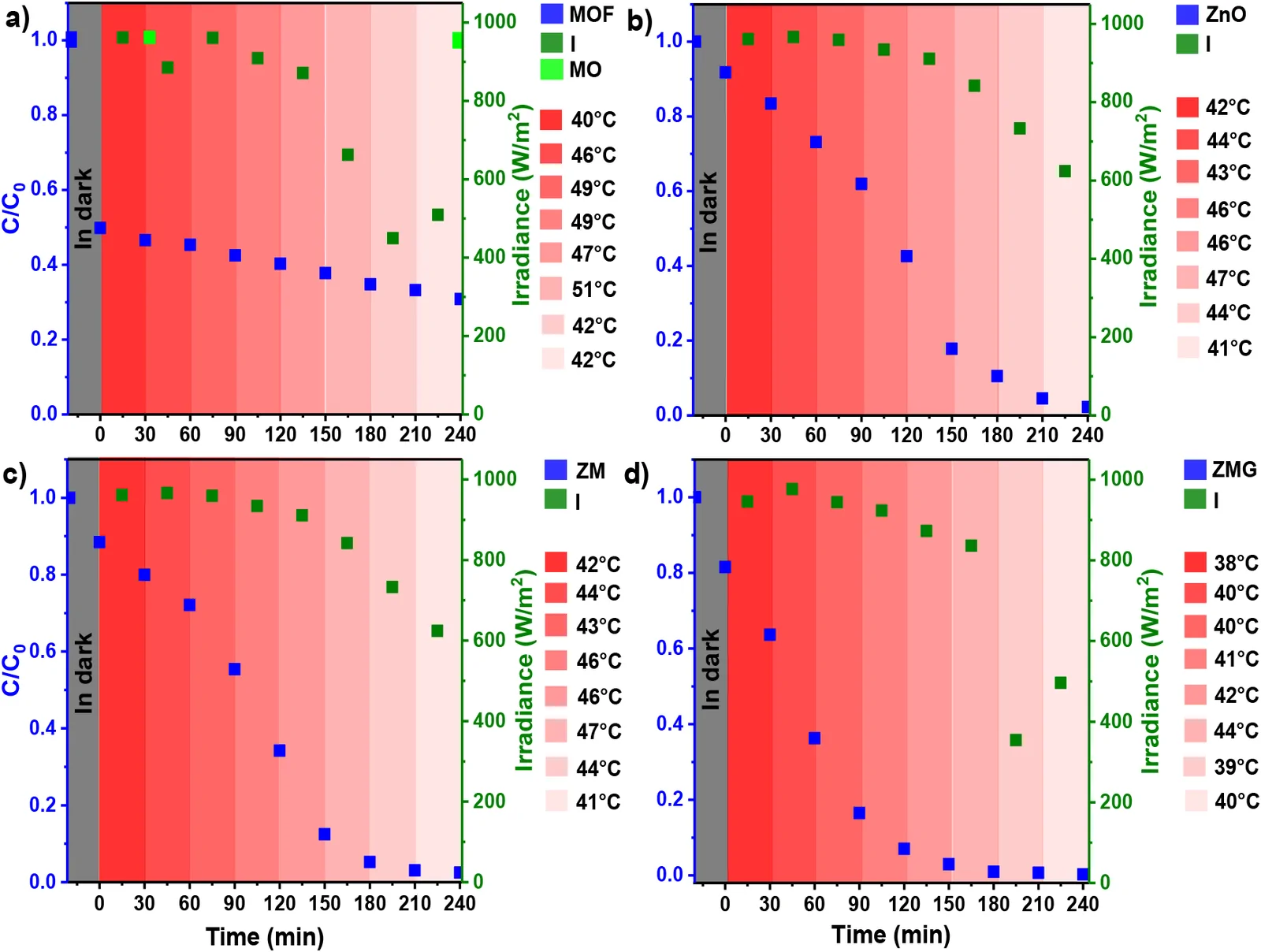
Photocatalytic degradation of MO under natural sunlight using a) MIL-101­(Fe), b) ZnO, c) ZM, and d) ZMG. The lime-green symbols in Figure 10a correspond to MO photolysis in the absence of a photocatalyst. The plots show the concentration of MO (C/C_0_, blue symbols) and solar irradiance (I, green symbols) as functions of time, with the corresponding temperature variation (red background). Irradiance values are expressed as average readings every 30 min.

The plots in Figure 10 also detail the environmental conditions recorded during the experiments. Irradiance (I), represented by green data points, reached values between 900 and 1000 W/m^2^ during the first 120 min of the test. These values are consistent with the typical solar profile in the city of Mérida during the spring season, within the peak-intensity interval (between 11:00 a.m. and 3:00 p.m.). These irradiance values are comparable to those obtained in controlled laboratory experiments using solar simulators.^59^ The most significant decrease in MO concentration occurs during this period, which will be analyzed in more detail later. Subsequently, irradiance decreased gradually, reflecting the expected diurnal behavior; minor deviations from this trend are attributed to isolated cloud passages during the measurements. The ambient temperature recorded by the pyranometer ranged from 38 to 51 °C and showed a direct correlation with solar irradiance during the first 120 min of the experiment. During the subsequent 60 min, the recorded ambient temperature remained elevated despite the gradual decrease in irradiance. This behavior reflects the thermal inertia of the surrounding outdoor environment, where the concrete rooftop gradually released the heat accumulated during peak solar irradiation. It is important to note that this effect corresponds to the ambient conditions surrounding the experiment rather than to direct heat transfer to the reaction vessels, which were placed on a laboratory magnetic stirrer (with the heating function switched off) positioned on a table approximately 1 m above the rooftop surface. Under these conditions, the control photolysis experiment showed no significant decrease in MO concentration, confirming that the observed MO removal was primarily associated with photocatalytic activity rather than thermal effects alone.

In the adsorption–desorption process, represented by the gray band in the plots in Figure 10, the MIL-101­(Fe) adsorbed approximately 50% of MO, attributable to its high surface area (384.33 m^2^/g) and porosity.^60^ In contrast, the ternary ZMG composite exhibited approximately 20% adsorption, despite having a larger surface area (484.41 m^2^/g). This apparent contradiction is explained by the combined effects of the reduced accessibility to adsorption sites after composite formation and the modified surface chemistry introduced by the GLC phase, including electrostatic interactions with the anionic MO molecules, which together limit the initial dye adsorption.^61^ In this case, adsorption is mainly governed by π–π stacking interactions between the aromatic rings of MO and the graphitic domains of GLC.^62^ However, this type of interaction is limited to the initial removal process; thus, incorporating ZnO and MIL-101­(Fe) into the GLC structure compensates for this limitation in the ZMG ternary composite. These semiconductors promote photoinduced charge separation, highlighting the complementary nature of adsorption and photodegradation processes.

Upon exposure to sunlight, the photocatalytic process begins. During the first 120 min of irradiation (900–1000 W/m^2^), the MIL-101­(Fe) achieved approximately 10% MO removal, despite exhibiting a high adsorption capacity in the dark of around 50% (Figure 10a). This behavior, characterized by high initial adsorption but low photocatalytic activity, has been previously reported for MIL-101­(Fe) with various molecules,^14,35,63^ confirming its limited efficiency when used as a standalone photocatalyst. The ZnO, on the other hand, achieved approximately 50% removal in the same period (Figure 10b), confirming its well-known photocatalytic activity under UV irradiation; however, its efficiency decreases under natural sunlight due to its limited absorption in the visible region.^64^

Regarding the composites, we can observe that the ZM composite displayed intermediate behavior, reaching about 60% MO removal within the same interval (Figure 10c). This improvement is attributed to the heterojunction formed between ZnO and MIL-101­(Fe), which enhances the efficient separation of photoinduced charges and extends the absorption range into the visible light region, thereby increasing the generation of reactive species responsible for MO oxidation and removal.^65^ Finally, the ternary ZMG material (Figure 10d) achieved approximately 85% MO removal within the first 90 min, showing a better correlation between photocatalytic performance and solar irradiance. Continued irradiation further increased the removal efficiency, reaching nearly complete MO removal after 240 min. This result confirms that its efficiency relies not only on structural and electronic properties but also on the synergistic relationship among MOF, ZnO, and GLC, which optimizes the photon flux under real irradiance conditions. Furthermore, after 240 min, the ZnO, ZM, and ZMG materials exhibited nearly complete disappearance of the characteristic MO absorption band, corresponding to nearly complete MO removal, despite a progressive decrease in irradiance, confirming their strong photocatalytic efficiency under natural sunlight. This result demonstrates that not only the initial reaction rate but also the ability to sustain high efficiency over extended periods is a key aspect for evaluating photocatalytic performance.

Using the Langmuir–Hinshelwood model, we evaluated the photodegradation kinetics of MO using a pseudo-first-order model eq 2. The high linearity of the ln­(C_0_/C) versus reaction time plots obtained for each catalyst (Figure 11a), with correlation coefficients (R^2^) close to 1, confirms the suitability of the model. This result is particularly relevant since the pseudo-first-order kinetics remained valid under the variable solar irradiance conditions of the experiment. Most of the MO removal occurred during the first 90–120 min, when solar irradiance remained close to its maximum value (approximately 900–1000 W/m^2^). Despite the decrease in light intensity after 120 min (Figure 10), degradation continued in proportion to the MO concentration. This result suggests that, even at reduced irradiance, the photon flux was sufficient to sustain the photocatalytic reaction, enabling efficient degradation even at low contaminant concentrations. The reaction rate constants (κ) calculated for MOF, ZnO, ZM, and ZMG were 0.0031 min^–1^, 0.016 min^–1^, 0.017 min^–1^, and 0.025 min^–1^, respectively. The ZMG composite exhibited the highest rate constant, approximately 8 times that of MOF, 1.5 times that of ZnO, and 1.4 times that of ZM. These results confirm the ZMG’s superior photocatalytic efficiency under solar irradiation and highlight the synergistic effect of its components, which significantly accelerates the reaction rate.

**Figure 11:**
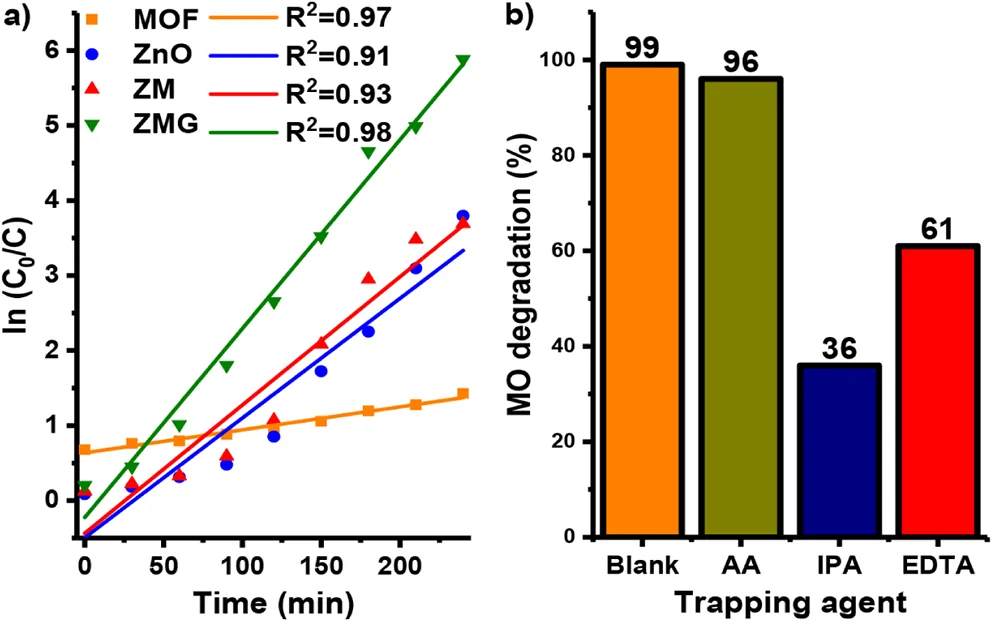
Kinetic analysis of the photocatalysts. a) pseudo-first-order kinetic plots of MO degradation under natural sunlight for MIL-101­(Fe), ZnO, ZM, and ZMG, and b) effect of scavengers on catalyst performance ZMG under sunlight.

To identify the active species responsible for ZMG’s high efficiency, we performed radical-scavenging experiments under solar irradiation (Figure 11b).^66,67^ The addition of isopropanol (IPA), a selective scavenger of hydroxyl radicals (•OH), drastically reduced MO degradation from 99% to 36%. This 63% loss suggests that •OH radicals are the predominant oxidative species under the present experimental conditions. With their high oxidation potential, •OH radicals effectively attack the aromatic structure and the azo bond of MO, initiating a series of oxidative reactions that lead to its decolorization and degradation.^68^ The addition of EDTA, a well-known h^+^ scavenger, also caused substantial inhibition: the degradation efficiency decreased from 99% to 61%, indicating that photogenerated holes play a significant role in the photocatalytic process. By scavenging h^+^, EDTA suppresses hole-mediated oxidation reactions and limits the formation of hydroxyl radicals, thereby reducing the overall photocatalytic efficiency. By neutralizing the holes, EDTA blocks both processes at their origins, demonstrating that valence-band holes are indispensable for a significant fraction of the oxidative activity.^69^ The addition of ascorbic acid (AA), a superoxide radical (•O_2_^–^) scavenger, did not significantly reduce photocatalytic activity. This indicates that •O_2_^–^ does not play a significant role as a direct oxidant in MO degradation. Although it is likely formed on the catalyst surface, its effect is secondary compared to the strong oxidizing power of •OH radicals and h^+^. Therefore, the role of •O_2_^–^ is mainly indirect, acting as an electron acceptor and helping reduce charge recombination.

The stability and reusability of a photocatalyst are critical parameters for practical applications, particularly when evaluating their performance under real environmental conditions. To assess the durability of the ZMG material, four consecutive photocatalytic cycles for MO removal were performed under natural sunlight, with an exposure time of 240 min per cycle (Figure 12). After each test, the catalyst was recovered by centrifugation, washed with distilled water, and dried at room temperature, following a standard procedure for reusability. The photocatalytic performance of ZMG remained consistently high throughout these cycles, with only a slight decrease in removal efficiency, from 99.8% in the first cycle to 96% in the fourth, demonstrating its excellent stability and functional integrity.

**Figure 12:**
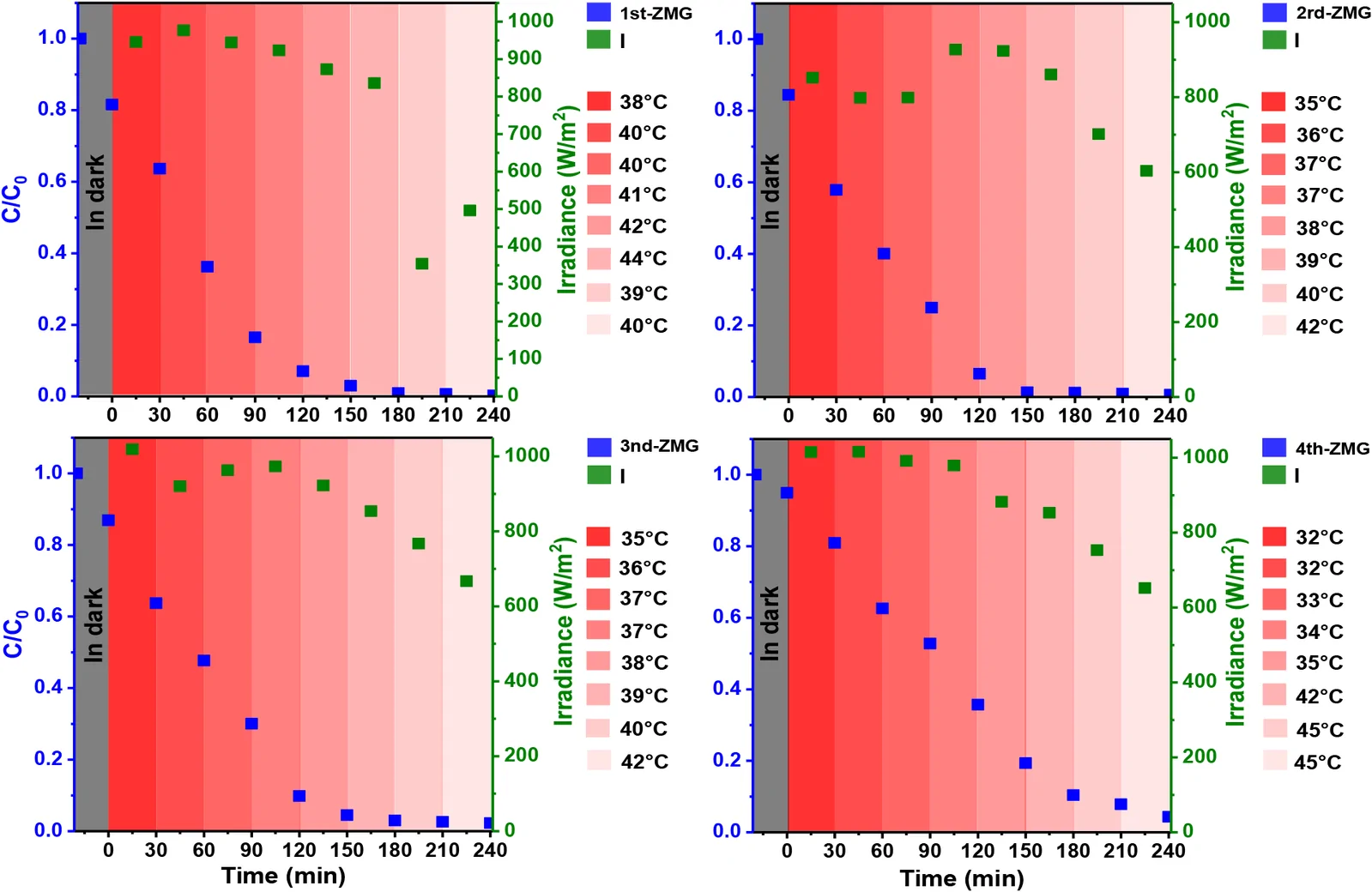
Stability tests for MO degradation using the ZMG compound under natural sunlight over four consecutive cycles. The plots show the concentration of MO (C/C_0_, blue symbols) and the solar irradiance (I, green symbols) as functions of time, with the corresponding temperature variation (red background). Irradiance values are expressed as average readings every 30 min.

The kinetic analysis based on the pseudo-first-order model (Figure 13a) revealed a gradual decrease in the rate constant κ, from 0.025 min^–1^ in the first cycle to 0.013 min^–1^ in the fourth cycle. This reduction in reaction rate is attributed to surface fouling, caused by the adsorption of MO molecules or intermediate oxidation products that partially block the catalyst’s active sites. By X-ray diffraction, we confirmed that the catalyst’s structural stability (Figure 13b) remained unchanged, with no significant differences from the initial material, indicating that the crystalline structure was preserved. The combination of high functional and structural stability, along with demonstrated efficiency under natural sunlight, validates the ZMG ternary photocatalyst as an emerging candidate for the photocatalytic treatment of dye-contaminated wastewater under real environmental conditions.

**Figure 13:**
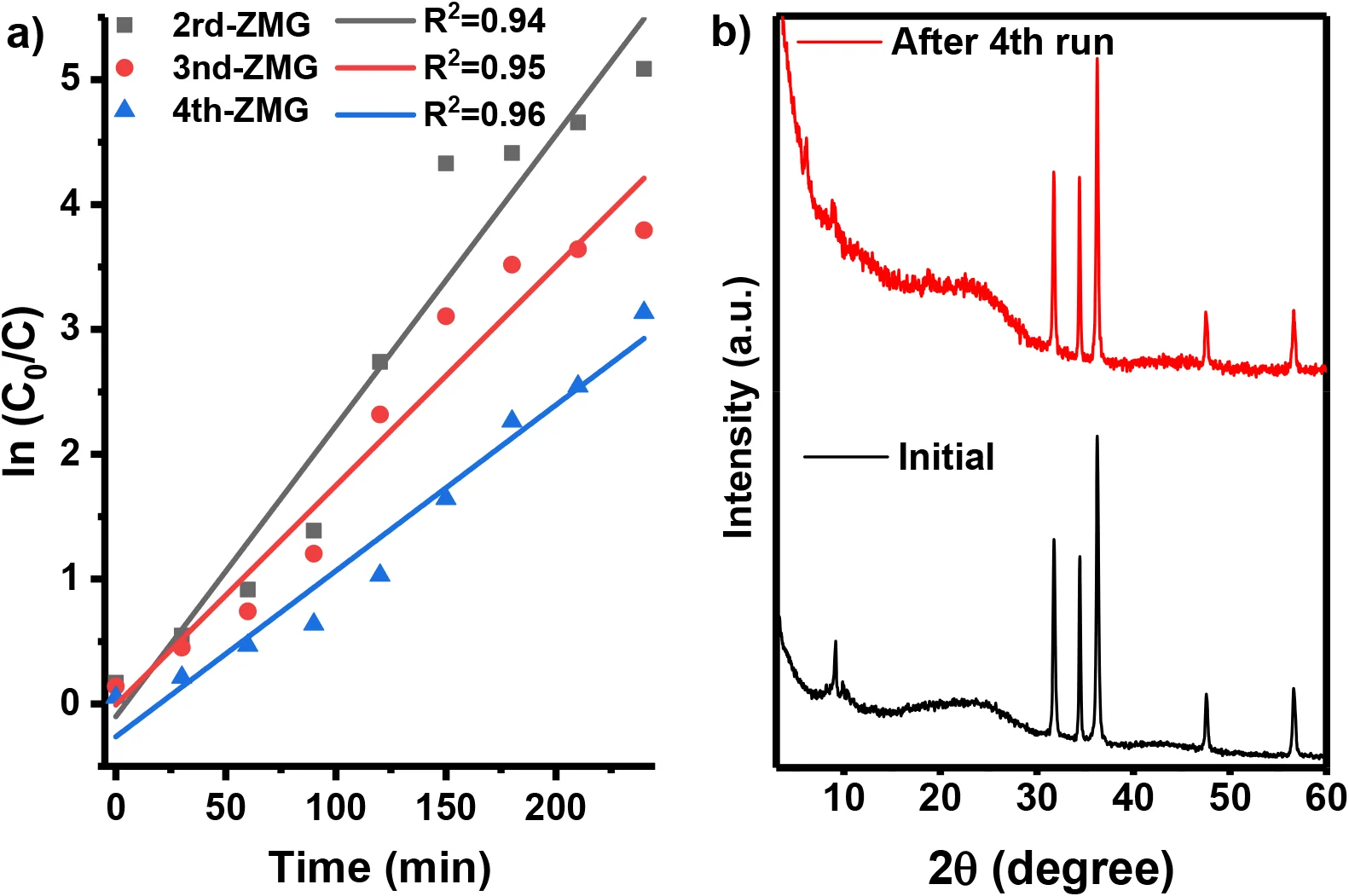
Kinetic analysis and structural stability of the photocatalysts. a) pseudo-first-order kinetic plots of ZMG during four consecutive reuse cycles, and b) X-ray diffractograms of the ZMG ternary photocatalyst before use (black line) and after the fourth photocatalytic cycle (red line), showing preservation of the crystalline structure.

Additionally, we tested the photocatalytic activity of our materials under laboratory conditions, using a UV irradiation source. In each experiment, we used a solution containing 10 mg of photocatalyst and 40 mL of MO at 40 ppm, with a 30 min presolution time to achieve adsorption–desorption equilibrium. The results (Figure 14a) show that under UV irradiation, the compounds’ photocatalytic activity increases, reaching more than 90% removal within 60 min of reaction. From the degradation data, we obtained the following rate constants (κ): 0.041 min^–1^ (MOF), 0.046 min^–1^ (ZnO), 0.051 min^–1^ (ZM), and 0.070 min^–1^ (ZMG). These values are higher than those obtained under sunlight, confirming that the type of light source affects the efficiency of the photocatalytic process. The ZMG ternary composite exhibits the highest rate constant (Figure 14b) under UV light (0.070 min^–1^), approximately three times higher than that obtained under solar light (0.025 min^–1^).

**Figure 14:**
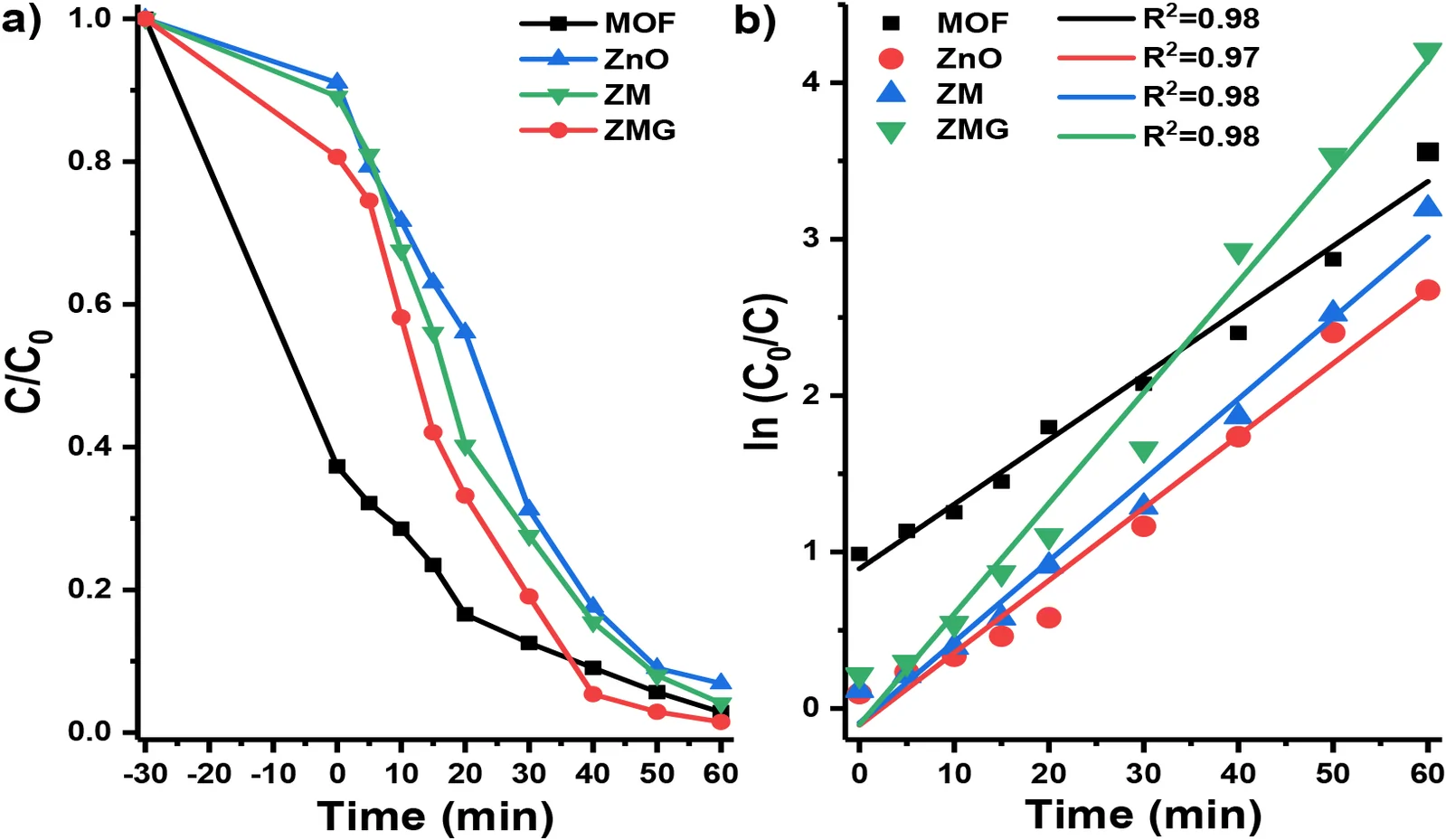
a) photocatalytic degradation of MO under UV irradiation using the MIL-101­(Fe), ZnO, ZM, and ZMG compounds, and b) first-order reaction kinetic curves for MO using the MIL-101­(Fe), ZnO, ZM, and ZMG compounds under UV irradiation.

The photodegradation results and their corresponding rate constants are summarized in Table 1 and illustrated in Figure 15. The analysis of these data strongly confirms the superior photocatalytic activity of the ZMG composite, which exhibits the highest rate constant (κ) under both natural sunlight and UV irradiation. Beyond this exceptional characteristic, the stability assessment shows that although the ZMG rate constant slightly decreases across multiple reuse cycles, it maintains efficient photocatalytic performance. This quantitative evidence demonstrates that the ZMG ternary composite is the most efficient and robust catalyst in our series, a key finding for its practical application in wastewater treatment. Although the photocatalytic performance under laboratory UV irradiation was higher than that under natural sunlight, this behavior is expected because UV irradiation provides a higher photon flux within the absorption range of ZnO under controlled conditions. In contrast, the sunlight experiments were performed under real outdoor conditions, where the UV fraction represents only a small portion of the solar spectrum and the irradiance continuously fluctuates. Therefore, the photocatalytic performance achieved under natural sunlight demonstrates the practical applicability of the ZMG composite for solar-driven wastewater treatment.

**Table 1:** Rate Constants from MO Degradation Studies

	Under sunlight				Under UV light			
Sample	**Time (min)**	**Photocatalytic removal under sunlight (%)**	**κ (min** ^**–1**^ **) sunlight**	**R** ^**2**^ **sunlight**	**Time (min)**	**Photocatalytic removal under UV light (%)**	**κ (min** ^**–1**^ **) UV light**	**R** ^**2**^ **UV light**
**MOF**	120	10	0.003	0.97	60	97	0.041	0.98
**ZnO**	120	50	0.016	0.91	60	93	0.046	0.97
**ZM**	120	60	0.017	0.93	60	96	0.051	0.98
**ZMG**	120	90	0.025	0.98	60	99	0.070	0.98

**Figure 15:**
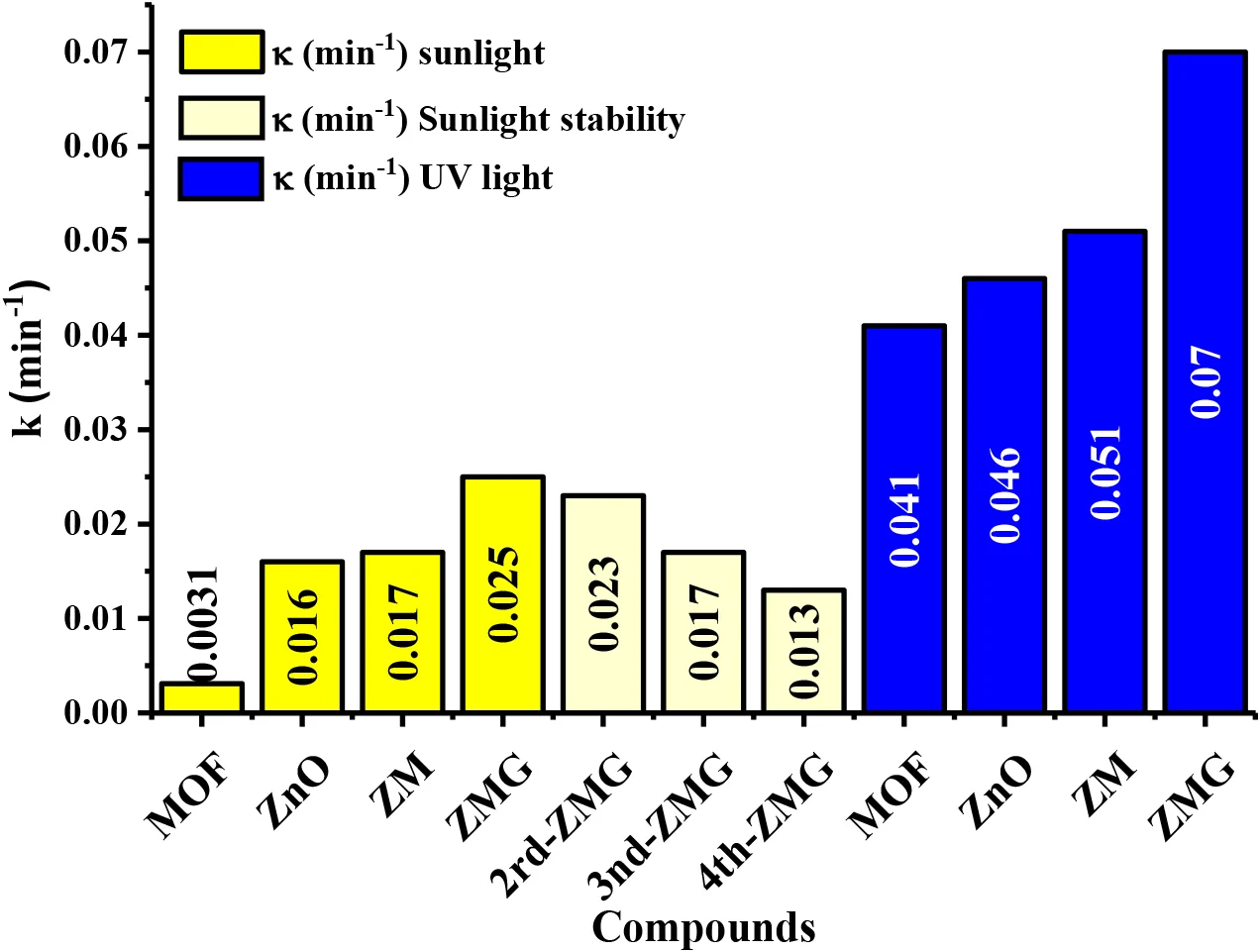
Comparison of the apparent rate constants (κ) for the photocatalysts under sunlight and UV irradiation, as well as after stability tests.

In Table 2, we present a direct comparison between the degradation results obtained in this study and those reported for other photocatalytic systems in the literature. Although there are variations in the initial experimental conditions, the data show that the degradation percentages obtained in the present work are higher than those reported in previous studies. In particular, higher degradation percentages are obtained at shorter reaction times or with lower photocatalyst amounts.

**Table 2:** Comparative Analysis of the MO Photodegradation Performance under Several Photocatalysts and Conditions

**Photocatalyst**	**Time (min)**	**MO concentration (ppm)**	**Volume (mL)**	**Photocatalyst quantity**	**Irradiation type**	**Degradation (%)**	**Ref**
Ag-ZnO	30	10	100	120 mg	Sunlight	100	^66^
FeMnO_3_@Ag	120	10	100	200 mg	Visible light	84.2	^67^
Ag@SiO_2_/TiO_2_	120	4.9	100.5	0.06 g	Simulated sunlight	97	^70^
TiO_2_/Biochar	270	50	100	0.6 g	Simulated sunlight	97.98	^71^
CdS	90	10	50	10 mg	Sunlight	85	^72^
Pt/TiO_2_	60	20		2.5 g/L	Visible light	90.26	^73^
CuO/MoS_2_@gCN	35	10	100	20 mg	UV light	85.14	^74^
Zn-MOF	200	10	50	125 mg	Simulated sunlight	82	^75^
Fe_2_O_3_/g-C_3_N_4_	60	10	100	100 mg	Visible-light	92.2	^76^
Fe_3_O_4_/GO	240	10	40	0.175 g	UV light	99.05	^77^
ZMG	90	10	40	10 mg	Sunlight	85	This Work
ZMG	60	10	40	10 mg	UV light	100	This Work

## 4 Discussion

The MO degradation results obtained under natural sunlight validate the central hypothesis of this study: the synergistic combination of ZnO, MIL-101­(Fe), and GLC significantly enhances photocatalytic performance. ZnO provides strong UV-driven activity, MIL-101­(Fe) extends absorption into the visible region, and GLC acts as an efficient electron acceptor. Together, these contributions promote faster charge separation, generate higher concentrations of reactive species (•OH and h^+^), and yield substantially improved catalytic turnover relative to the individual components. Additionally, the high surface area of GLC enhances dye adsorption, increasing the frequency of contaminant–catalyst interactions.

Kinetic analysis confirmed that the ZMG composite exhibits the highest rate constant (0.025 min^–1^), markedly surpassing ZnO and MIL-101­(Fe). Radical-trapping experiments revealed that •OH radicals are the dominant oxidants, with valence-band h^+^ contributing significantly to MO degradation. The negligible effect of ascorbic acid indicates that superoxide (•O_2_^–^) plays only a minor role, consistent with the expected band energetics.

To rationalize the enhanced performance, a band-alignment model was considered using literature values. ZnO presents CB and VB edges at approximately −0.2 eV and +2.8 eV vs NHE, respectively,^78–81^ while MIL-101­(Fe) typically exhibits a CB near −0.1 eV and a VB around +2.3 eV.^14,35,38,43^ This configuration corresponds to a Type-I band alignment in which both the CB and VB of MIL-101­(Fe) lie within the ZnO bandgap.^82^ Under illumination, electrons photogenerated in ZnO transfer downhill to the slightly lower CB of MIL-101­(Fe) and subsequently to GLC.^83,84^ This cascade migration suppresses recombination, consistent with the kinetic results and the observed scavenger behavior.

The VB position of ZnO (+2.8 eV) is sufficiently positive to oxidize OH^–^ to •OH ((OH^–^/•OH) ≈ +2.40 V vs NHE) and also H_2_O to •OH ((H_2_O/•OH) ≈ +2.73 eV vs NHE).^85^ This explains why •OH radicals emerge as the predominant oxidative species in the ZMG system. Conversely, the limited participation of •O_2_^–^ correlates with CB potentials only slightly more negative than the O_2_/•O_2_^–^ redox potential (≈ −0.33 V vs NHE), enabling partial superoxide formation but favoring electron retention on GLC rather than efficient O_2_ reduction pathways.

Durability is also essential for practical application. The ZMG composite retained high efficiency over multiple cycles with minimal loss of activity, and XRD confirmed its structural stability. Its strong performance under both natural sunlight and UV irradiation indicates that the enhanced activity results from an intrinsically favorable charge-transfer architecture rather than an illumination-specific effect. Overall, the integration of ZnO, MIL-101­(Fe), and biomass-derived GLC forms a sustainable heterostructure with efficient charge separation and strong oxidative capability, making ZMG a promising photocatalyst for wastewater remediation under real environmental conditions.

As a possible mechanism for MO degradation by the ZMG ternary composite photocatalyst, we propose the reaction sequence shown in eqs 4–13. According to the results obtained, under sunlight irradiation, ZnO absorbs photons, promoting e^–^ from valence band (VB) to conduction band (CB), generating e^–^–h^+^ pairs (eq 4). Since MIL-101­(Fe) has a lower E_g_, electrons in CB_ZnO_ are transferred to CB_MIL‑101(Fe)_, favoring charge separation and inhibiting recombination (eq 5). Similarly, MIL-101­(Fe) also generates additional e^–^–h^+^ pairs under irradiation (eq 6). GLC acts as an efficient electron collector, facilitating the transport of electrons from both CB_MIL‑101(Fe)_(e^–^) and CB_ZnO_(e^–^) to its surface (eqs 7–8) owing to its two-dimensional π-conjugated structure and high electronic mobility.^84^ These electrons can react with dissolved molecular oxygen (O_2_) to generate superoxide radicals (•O_2_^–^) (eq 9). However, according to the radical-scavenging experiments, the contribution of •O_2_^–^ to MO oxidation is secondary, indicating that these radicals mainly participate in electron consumption and charge-separation processes rather than acting as the predominant oxidizing species. In contrast, the photogenerated holes in VB_ZnO_(h^+^) and VB_MIL‑101(Fe)_(h^+^) react with water molecules to produce hydroxyl radicals (•OH) (eqs 10–11), which were identified as the dominant reactive species based on the pronounced inhibition observed after IPA addition. The contribution of h^+^ was further confirmed by the EDTA scavenging experiments, demonstrating that valence-band holes play an essential role in the generation of •OH radicals, either through the oxidation of water or hydroxide ions (eq 12). These hydroxyl radicals subsequently attack the MO molecules, leading to their decolorization and oxidative degradation into smaller oxidation products (eq 13).^86^ The proposed reaction mechanism for radical generation and subsequent MO degradation by the ZMG photocatalyst under sunlight irradiation is shown in Figure 16.

4 $$ZnO+hv\to {\left(e^{-}+h^{+}\right)}_{ZnO}$$

5 $$ZnO\left(e^{-}\right)+MI\mathrm{L‐}101\left(Fe\right)\to CB_{MI\mathrm{L‐}101\left(Fe\right)}\left(e^{-}\right)$$

6 $$MI\mathrm{L‐}101\left(Fe\right)+hv\to {\left(e^{-}+h^{+}\right)}_{MI\mathrm{L‐}101\left(Fe\right)}$$

7 $$CB_{MI\mathrm{L‐}101\left(Fe\right)}\left(e^{-}\right)+GLC\to GLC\left(e^{-}\right)$$

8 $$CB_{ZnO}\left(e^{-}\right)+GLC\to GLC\left(e^{-}\right)$$

9 $$GLC\left(e^{-}\right)+O_{2}\to •{O_{2}}^{-}$$

10 $$VB_{ZnO}\left(h^{+}\right)+H_{2}O\to •\mathrm{OH}+H^{+}$$

11 $$VB_{MI\mathrm{L‐}101\left(Fe\right)}\left(h^{+}\right)+H_{2}O\to •\mathrm{OH}+H^{+}$$

12 $$OH^{-}+h^{+}\to •\mathrm{OH}$$

13 •OH+MO→Degradation

**Figure 16:**
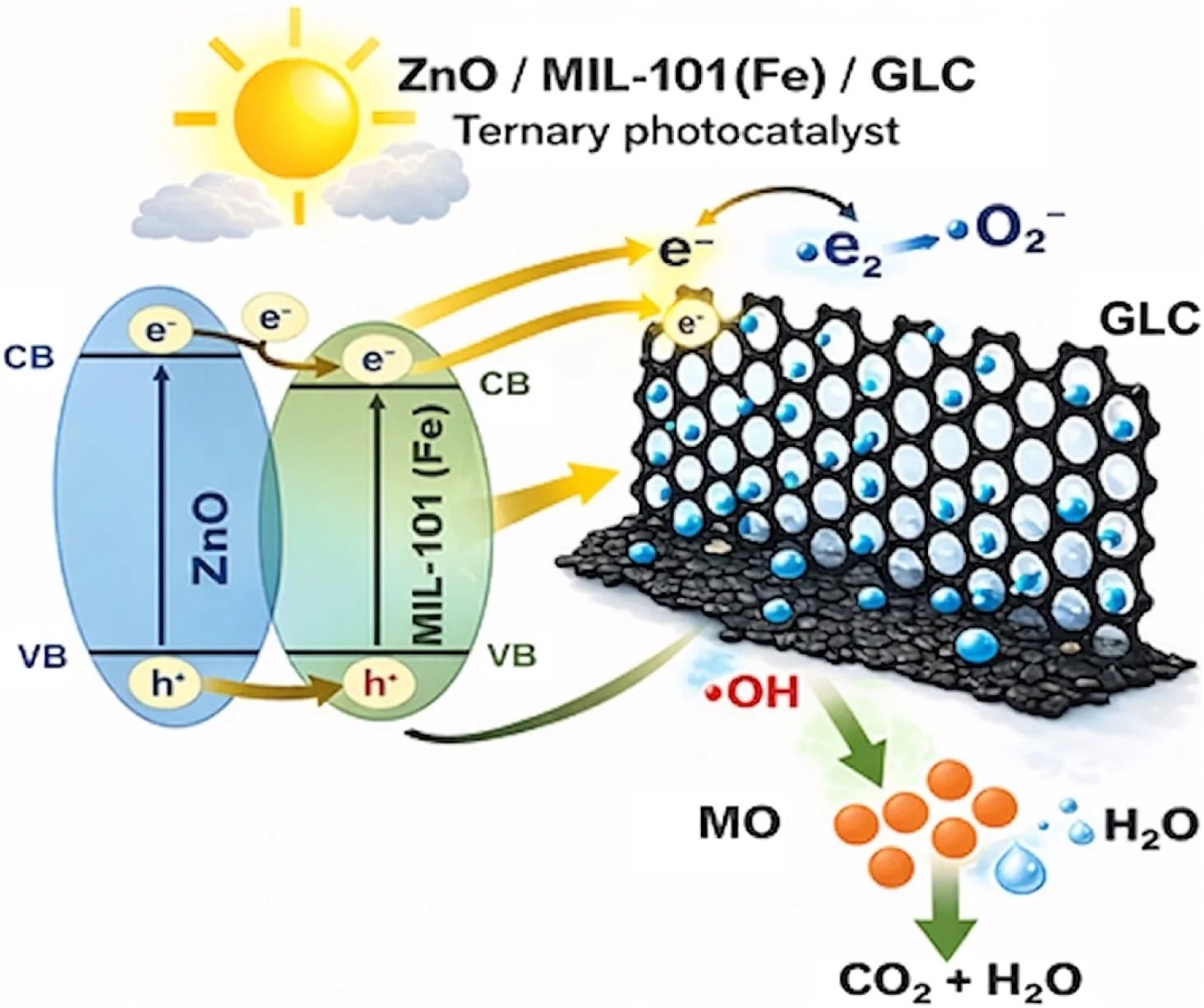
Schematic illustration of the photocatalytic mechanism of ZMG under sunlight irradiation, showing charge separation in ZnO and MIL-101­(Fe), electron transfer to GLC, and formation of reactive oxygen species.

## 5 Conclusions

In this work, we successfully demonstrated a sustainable and efficient strategy for photocatalyst design by synthesizing the novel ternary composite ZnO-MIL-101­(Fe)/GLC (ZMG) from the valorization of the invasive biomass *Phragmites australis*. The ZMG system validated the synergetic hypothesis by exhibiting superior photocatalytic efficiency under natural sunlight, achieving 85% MO removal within the first 90 min and nearly complete MO removal after 240 min. Under laboratory UV irradiation, the ZMG demonstrated superior photocatalytic kinetics, achieving complete MO removal within 60 min and yielding the highest rate constant (κ = 0.070 min^–1^), nearly 3 times higher than that obtained under natural sunlight. This remarkable enhancement was confirmed to be driven by optimized charge separation within the ZMG heterostructure, where GLC plays a key role, favoring the action of high-potential oxidants (•OH and h^+^), thereby promoting the efficient photocatalytic degradation of MO. Beyond its high performance, the ZMG demonstrated excellent structural stability and reusability, maintaining a degradation efficiency above 96% after four consecutive cycles. Furthermore, its ability to achieve near-complete MO removal under declining solar irradiance underscores its sustained efficiency and viability in variable-sunlight environments. These findings establish a promising precedent for integrating residual biomass into high-performance catalysts and position the ZMG as a robust, kinetically efficient candidate for practical wastewater remediation.

## Data Availability

Data will be made available upon request.

## References

[1] Gaber M. M., Shokry H., Samy M. A., El-Bestawy E. (2024). Green Approach
for Fabricating Hybrids of Food Waste-Derived Biochar/zinc Oxide for
Effective Degradation of Bromothymol Blue Dye in a Photocatalysis/persulfate
Activation System. Chemosphere.

[2] Radičić R., Jurov A., Zavašnik J., Kovač J., Brusar V., Vdović S., Novko D., Krstulović N. (2024). UV and Solar-Driven
Photocatalysis of Organic Dyes Using ZnO-Ag Heterojunction Nanoparticles
Synthesized by One-Step Laser Synthesis in Water. Appl. Surf. Sci.

[3] Liu T., Qian J., Wang C., Wang Y., Yang Y., Kong B., Qian Y., Wu W. (2020). Enhanced Photocatalytic
Degradation Performance of Mono-Disperse ZnS Nano-Flake on Biocarbon
Sheets. Inorg. Chem. Commun.

[4] Regadera-Macías A. M., Morales-Torres S., Pastrana-Martínez L. M., Maldonado-Hódar F. J. (2023). Ethylene
Removal by Adsorption and Photocatalytic Oxidation Using Biocarbon–TiO_2_ Nanocomposites. Catal. Today.

[5] Wu Z., Zhu Z., Ma J., Zhou M., Wu Z., You H., Zhang H., Li N., Wang F. (2024). High Piezo-Photocatalysis
of BaTiO_3_ Nanofibers for Organic Dye Decomposition. Surf. Interfaces.

[6] Byrne C., Subramanian G., Pillai S. C. (2018). Recent Advances
in Photocatalysis
for Environmental Applications. J. Environ.
Chem. Eng.

[7] Alhumaimess M. S. (2020). Metal–organic
Frameworks and Their Catalytic Applications. J. Saudi Chem. Soc.

[8] Chen D., Zheng Y.-T., Huang N.-Y., Xu Q. (2024). Metal-Organic
Framework
Composites for Photocatalysis. EnergyChem.

[9] Xu F., Zhang P., Navrotsky A., Yuan Z.-Y., Ren T.-Z., Halasa M., Su B.-L. (2007). Hierarchically
Assembled Porous ZnO
Nanoparticles: Synthesis, Surface Energy, and Photocatalytic Activity. Chem. Mater.

[10] Onkani S. P., Akpotu S. O., Diagboya P. N., Mtunzi F., Osabohien E. (2024). Enhanced Aqueous
Photo-Degradation of Ciprofloxacin and Tetracycline Using Ag-Decorated
ZnO Plasmonic Catalyst. ACS Sustain. Resour.
Manag.

[11] Dhongade S., Koinkar P., Katayama T., Maki Y., Furube A. (2021). Charge Separation
Dynamics in In_2_Se_3_/ZnO/Au Ternary System for
Enhanced Photocatalytic Degradation of Methylene Blue under Visible
Light. J. Photochem. Photobiol., A.

[12] Sawate A., Kumar G., Patel M., Sathe B. R., Katayama T., Yeetsorn R., Furube A., Koinkar P. (2026). Investigating the photocatalytic
performance of ternary nanocomposite Pd/rGO/MoO_3_ under
visible light. J. Alloys Compd.

[13] Fogaça L. Z., Vicentini J. C. M., de Freitas C. F., Braga T. L., da Silva F. A., de Souza M., Baesso M. L., Caetano W., Batistela V. R., Scaliante M. H. N. O. (2023). Nanocomposites
of Nb_2_O_5_ and ZnO
with Reduced Graphene Oxide for Heterogeneous Photocatalysis of Dyes. Catal. Commun.

[14] Zhu J., Zhou H., Wang R., Wang C. (2022). Core-Shell nanosheets@MIL-101­(Fe)
Heterostructures with Enhanced Photocatalytic Activity Promoted by
Peroxymonosulfate. J. Taiwan Inst. Chem. Eng.

[15] Swaminaathan P., Saravanan A., Yaashikaa P. R., Vickram A. S. (2024). Recent Advances
in Photocatalytic Degradation of Persistent Organic Pollutants: Mechanisms,
Challenges, and Modification Strategies. Sustain.
Chem. Environ.

[16] John D., Jose J., Bhat S. G., Achari V. S. (2021). Integration of Heterogeneous
Photocatalysis and Persulfate Based Oxidation Using TiO_2_-Reduced Graphene Oxide for Water Decontamination and Disinfection. Heliyon.

[17] Immanuvel A., Pandiyarajan C., Sivaranjana P., Kandasamy M., Rameshkumar P. (2025). Effective Quenching of Charge Recombination
in ZnO
Nanorods Supported Reduced Graphene Oxide for Improved Photocatalysis. Inorg. Chem. Commun.

[18] Wen H. M., Jin H., Pan J., Hong Q., Lv H., Xie B., Xiao J. Q., Hu J. (2022). Hollow FeNi/NiFe_2_O_4_-Codoped Carbon Composite
Nanorods for Electromagnetic Wave
Absorption. ACS Appl. Nano Mater.

[19] Lindsay D. L., Freeland J., Gong P., Guan X., Harms N. E., Kowalski K. P., Lance R. F., Oh D.-H., Sartain B. T., Wendell D. L. (2023). Genetic Analysis
of North American *Phragmites
Australis* Guides Management Approaches. Aquat. Bot.

[20] Bagşaran
Kankılıç G., Metin A. (2020). *Phragmites
Australis* as a New Cellulose Source: Extraction, Characterization
and Adsorption of Methylene Blue. J. Mol. Liq.

[21] Pareja-Rodríguez R., Torres-García E., Martínez-Flores R., Rodríguez-Gattorno G. (2025). The Role of
Autogenous Atmosphere during Graphene-like Materials Formation from
Biomass: A Case Study from *Phragmites Australis*,
Thermokinetic Study and Structural Evolution. Mater. Today Commun.

[22] Lin Y. C., Kurniawan A., Valinton J. A. A., Chen C. H., Chuang S. H. (2024). Reduced
Graphene oxide–MoO_3_ Composites via Microwave-Assisted
Synthesis for Dual-Functional Photocatalysis of Organic Dyes and Heavy
Metal Cation under Simulated Sunlight Irradiation. J. Taiwan Inst. Chem. Eng.

[23] Ramos-Corona A., Rangel R., Quintana P., Alvarado-Gil J. J., Franco-Bacca A. P., Chan-Espinoza J. A., Ramos-Carrazco A., García R. (2024). Tunning the Structural and Photocatalytic Properties
of Nitrogen-Doped ZnSe/GO Compounds through Factorial Designs. J. Phys. Chem. C.

[24] Seehra M. S., Narang V., Geddam U. K., Stefaniak A. B. (2017). Correlation
between X-Ray Diffraction and Raman Spectra of 16 Commercial Graphene–based
Materials and Their Resulting Classification. Carbon.

[25] Joseph A., Yelekar G. R., Vijayanandan A. (2024). Efficiency of Titanium Dioxide-Reduced
Graphene Oxide in Carbamazepine Removal: A Comparative Study of Adsorption,
Photocatalysis, and Ultrafiltration Techniques. Appl. Catal. O: Open.

[26] Edebali S. (2023). Synthesis
and Characterization of MIL-101 (Fe) as Efficient Catalyst for Tetracycline
Degradation by Using NaBH_4_ Artificial Neural Network Modeling. Appl. Surf. Sci. Adv.

[27] Liu Z., He W., Zhang Q., Shapour H., Bakhtari M. F. (2021). Preparation of a
GO/MIL-101­(Fe) Composite for the Removal of Methyl Orange from Aqueous
Solution. ACS Omega.

[28] Fattahi M., Niazi Z., Esmaeili F., Mohammadi A. A., Shams M., Nguyen Le B. (2023). Boosting the
Adsorptive and Photocatalytic
Performance of MIL-101­(Fe) against Methylene Blue Dye through a Thermal
Post-Synthesis Modification. Sci. Rep.

[29] Amdeha E., Mohamed R. S. (2021). A Green Synthesized
Recyclable ZnO/MIL-101­(Fe) for
Rhodamine B Dye Removal via Adsorption and Photo-Degradation under
UV and Visible Light Irradiation. Environ. Technol.

[30] Vardhan
Patel R., Yadav A. (2022). Photocatalytic MIL101­(Fe)/ZnO Chitosan
Composites for Adsorptive Removal of Tetracycline Antibiotics from
the Aqueous Stream. J. Mol. Struct.

[31] Günduğar K., Semerci F. (2022). Compositing
of MIL-101­(Fe) with Reduced Graphene Oxide
and Polyaniline for Capacitive Energy Storage. Mater. Chem. Phys.

[32] Wu G., Liu Q., Ma L., Tian L., Ran J., Pan Y., Han J., Xing W. (2023). The Promoted Organic Pollutant and Visible-Light-Driven
Photocatalytic Degradation Efficiency of MIL-101­(Fe)/Bi_2_WO_6_ Z-Scheme Heterojunction Assisting and Mechanism. Colloids Surf., A.

[33] Chen H., Liu Y., Cai T., Dong W., Tang L., Xia X., Wang L., Li T. (2019). Boosting Photocatalytic Performance
in Mixed-Valence MIL-53­(Fe) by Changing Fe^II^/Fe^III^ Ratio. ACS Appl. Mater. Interfaces.

[34] Tang J., Wang J. (2018). Metal Organic Framework with Coordinatively Unsaturated Sites as
Efficient Fenton-like Catalyst for Enhanced Degradation of Sulfamethazine. Environ. Sci. Technol.

[35] Shi W., Fu Y., Sun H., Sun X., Hao C., Guo F., Tang Y. (2022). Construction of 0D/3D
CoFe_2_O_4_/MIL-101­(Fe) Complement
Each Other S-Scheme Heterojunction for Effectively Boosted Photocatalytic
Degradation of Tetracycline. Inorg. Chem. Commun.

[36] Jin Y., Mi X., Qian J., Ma N., Dai W. (2022). Modular Construction
of an MIL-101­(Fe)@MIL-100­(Fe) Dual-Compartment Nanoreactor and Its
Boosted Photocatalytic Activity toward Tetracycline. ACS Appl. Mater. Interfaces.

[37] Salman F., Zengin A., Çelik
Kazici H. (2020). Synthesis and Characterization of
Fe_3_O_4_-Supported Metal–organic Framework
MIL-101­(Fe) for a Highly Selective and Sensitive Hydrogen Peroxide
Electrochemical Sensor. Ionics.

[38] Zhao F., Liu Y., Hammouda S. B., Doshi B., Guijarro N., Min X., Tang C. J., Sillanpää M., Sivula K., Wang S. (2020). MIL-101­(Fe)/g-C_3_N_4_ for Enhanced Visible-Light-Driven
Photocatalysis toward Simultaneous Reduction of Cr­(VI) and Oxidation
of Bisphenol A in Aqueous Media. Appl. Catal.,
B.

[39] Kwan Y. C. G., Ng G. M., Huan C. H. A. (2015). Identification
of Functional Groups
and Determination of Carboxyl Formation Temperature in Graphene Oxide
Using the XPS O 1s Spectrum. Thin Solid Films.

[40] Herrera-Zamora D. M., Pareja-Rodríguez R., Ruiz-Gómez M., González-Gómez W. S., Velasco-Arias D., García-Valladares O., Escobar S., Rodríguez-Gattorno G., Martínez-Flores R. (2023). Dispersibility
Study of GO-like Biocarbons
Obtained from the Thermal Decomposition of Biomass: *Phragmites
Australis* and Carya Illinoinensis. Biomass Bioenergy.

[41] Hsieh S. H., Ting J. M. (2018). Characterization and Photocatalytic Performance of
Ternary Cu-Doped ZnO/Graphene Materials. Appl.
Surf. Sci.

[42] Chen L.-C., Tu Y.-J., Wang Y.-S., Kan R.-S., Huang C.-M. (2008). Characterization
and Photoreactivity of N-, S-, and C-Doped ZnO under UV and Visible
Light Illumination. J. Photochem. Photobiol.,
A.

[43] Hajiali M., Farhadian M., Tangestaninejad S., Khosravi M. (2022). Synthesis and Characterization
of Bi_2_MoO_6_/MIL-101­(Fe) as a Novel Composite
with Enhanced Photocatalytic Performance: Effect of Water Matrix and
Reaction Mechanism. Adv. Powder Technol.

[44] Khan M. E., Khan M. M., Cho M. H. (2016). CdS-Graphene
Nanocomposite for Efficient
Visible-Light-Driven Photocatalytic and Photoelectrochemical Applications. J. Colloid Interface Sci.

[45] Klein J., Kampermann L., Mockenhaupt B., Behrens M., Strunk J., Bacher G. (2023). Limitations
of the Tauc Plot Method. Adv. Funct. Mater.

[46] Bergman L., Chen X.-B., Huso J., Morrison J. L., Hoeck H. (2005). Raman Scattering
of Polar Modes of ZnO Crystallites. J. Appl.
Phys.

[47] Zhuo R. F., Feng H. T., Liang Q., Liu J. Z., Chen J. T., Yan D., Feng J. J., Li H. J., Cheng S., Geng B. S., Xu X. Y., Wang J., Wu Z. G., Yan P. X., Yue G. H. (2008). Morphology-Controlled
Synthesis, Growth Mechanism,
Optical and Microwave Absorption Properties of ZnO Nanocombs. J. Phys. D: Appl. Phys.

[48] Chen Q., Huang H., Li X., Pan L., Chen Y., Zhong Q., Wu X., Yang S.-T. (2024). Raman Spectroscopic
Quantification of Graphene Oxide in Soil: Transport, Surficial Enrichment
and Environmental Effects. Environ. Pollut.

[49] Kong F., Tang B. (2023). Surface-Enhanced Raman
Scattering of Graphene Oxide Chemically Modified
by Perylene Diimide. Vib. Spectrosc.

[50] Krishnamoorthy K., Veerapandian M., Yun K., Kim S.-J. (2013). The Chemical and
Structural Analysis of Graphene Oxide with Different Degrees of Oxidation. Carbon.

[51] Eda G., Chhowalla M. (2010). Chemically Derived Graphene Oxide: Towards Large-Area
Thin-Film Electronics and Optoelectronics. Adv.
Mater.

[52] Abdelghani G. M., Ahmed A. B., Al-Zubaidi A. B. (2022). Synthesis, Characterization, and
the Influence of Energy of Irradiation on Optical Properties of ZnO
Nanostructures. Sci. Rep.

[53] Smal I. M., Yu Q., Veneman R., Fränzel-Luiten B., Brilman D. W. F. (2014). TG-FTIR
Measurement of CO_2_-H_2_O Co-Adsorption for CO_2_ Air Capture Sorbent Screening. Energy
Procedia.

[54] Rochman R. A., Wahyuningsih S., Ramelan A. H., Hanif Q. A. (2019). Preparation of Nitrogen
and Sulphur Co-Doped Reduced Graphene Oxide (rGO-NS) Using N and S
Heteroatom of Thiourea. IOP Conf Ser. Mater.
Sci. Eng.

[55] Mafamadi M., Etale A., Daramola M. O. (2024). Synthesis
and Characterization of
Graphene Oxide Functionalized with Dicarboxylic Acids. Mater. Today Proc.

[56] Allah M. A. A. H., Ibrahim H. K., Alshamsi H. A. (2025). Enhanced Adsorption,
Anticancer and
Antibacterial Potentials of Pontederia Crassipes L. Extract Mediated
Ecofriendly Synthesized ZnO/biochar Nanohybrid. Inorg. Chem. Commun.

[57] Lims S. C., Jose M., Aswathappa S., Jude Dhas S. S., Kumar R. S., Pham P. V. (2025). Exploring the Photocatalytic Breakdown
of Organic Pollutants Using Intercalated ZnO/SiO_2_ Nanocomposites. Mater. Chem. Phys.

[58] Jarrah A., Farhadi S. (2020). Encapsulation of K_6_P_2_W_18_O_62_ into Magnetic Nanoporous Fe_3_O_4_/MIL-101 (Fe) for Highly Enhanced Removal of Organic Dyes. J. Solid State Chem.

[59] García-Peña N. G., Nabil M., Pourjafari D., Peralta-Domínguez D., Padrón-Hernández W. Y., Franco-Bacca A. P., Ríos-Flores A., Heredia-Cervera B. E., Escalante R., Rodríguez Gattorno G., Acosta M., Pistor P., Anta J. A., Oskam G. (2025). Improving the Performance of Carbon-Based
Perovskite Solar Cells by the Incorporation of a Screen-Printed NiCo_2_O_4_ Interlayer. ACS Appl.
Energy Mater.

[60] Tang Y., Yin X., Mu M., Jiang Y., Li X., Zhang H., Ouyang T. (2020). Anatase TiO_2_@MIL-101­(Cr) Nanocomposite for
Photocatalytic Degradation of Bisphenol A. Colloids
Surf. A.

[61] Wang S., Dou J., Zhang T., Li S., Chen X. (2023). Selective Adsorption
of Methyl Orange and Methylene Blue by Porous Carbon Material Prepared
From Potassium Citrate. ACS Omega.

[62] Molla A., Li Y., Mandal B., Kang S. G., Hur S. H., Chung J. S. (2019). Selective
Adsorption of Organic Dyes on Graphene Oxide: Theoretical and Experimental
Analysis. Appl. Surf. Sci.

[63] He L., Dong Y., Zheng Y., Jia Q., Shan S., Zhang Y. (2019). A Novel Magnetic MIL-101­(Fe)/TiO_2_ Composite for Photo
Degradation of Tetracycline under Solar Light. J. Hazard. Mater.

[64] Raizada P., Sudhaik A., Singh P. (2019). Photocatalytic Water
Decontamination
Using Graphene and ZnO Coupled Photocatalysts: A Review. Mater. Sci. Energy Technol.

[65] Mahmoudi F., Bachas L. G. (2024). Application of Metal–Organic
Framework-Based
Composite Materials for Photodegradation of Dye Pollutants in Wastewater. Water.

[66] Stanley S., Jebasingh J. A., S M. V., Stanley P. K., Ponmani P., Shekinah M. E., Vasanthi J. (2021). Excellent Photocatalytic Degradation
of Methylene Blue, Rhodamine B and Methyl Orange Dyes by Ag-ZnO Nanocomposite
under Natural Sunlight Irradiation. Optik.

[67] Ahmad N., Khan M. R., Velu K. S., Mohandoss S. (2025). Development
of FeMnO_3_@Ag Perovskite via Sol-Gel Combustion for Superior
Photocatalytic Degradation of Methyl Orange under Visible Light Irradiation. Mater. Chem. Phys.

[68] Spadaro J. T., Isabelle L., Renganathan V. (1994). Hydroxyl Radical Mediated Degradation
of Azo Dyes: Evidence for Benzene Generation. Environ. Sci. Technol.

[69] Stanley R., Balu S. K., Alphas
Jebasingh J., Manisha Vidyavathy S. (2023). Super-Efficient
Photocatalytic Degradation of Methylene Blue, Methyl Orange and Rhodamine
B Dyes Using Low-Cost ZnO–MgO Nanocomposite under Natural Sunlight
and Its Bactericidal Activity. Res. Chem. Intermed.

[70] Zuo X., Zou S., Wu J., Ding B., Jiang A. (2024). Simulated Sunlight-Driven
Photocatalytic Activation of Peroxydisulfate by Core–shell
Ag@SiO_2_/TiO_2_ Nanocomposite for Efficient Methyl
Orange Degradation. Desalin. Water Treat.

[71] Liang S., An M., Xia S., Zhang B., Xue B., Xu G. (2023). Enhanced Photocatalytic
Degradation of Methyl Orange by TiO_2_/biochar Composites
under Simulated Sunlight Irradiation. Opt. Mater.

[72] Hojjati-Najafabadi A., Farahbakhsh E., Gholamalian G., Feng P., Davar F., Aminabhavi T. M., Vasseghian Y., Kamyab H., Rahimi H. (2024). Controllable
Synthesis of Nanostructured Flower-like Cadmium Sulfides for Photocatalytic
Degradation of Methyl Orange under Different Light Sources. J. Water Process. Eng.

[73] Thi
Thu Nhu V., Nguyen V.-T. (2025). Pt/TiO_2_ Nanoparticles
Synthesized via Gamma Irradiation to Improve Photocatalytic Degradation
of Methyl Orange in Visible Light. Nucl. Instruments
Methods Phys. Res. Sect. B.

[74] Alomayrah N., Ikram M., Alomairy S., Al-Buriahi M. S., Naziruddin Khan M., Farooq Warsi M., Irshad A. (2024). Fabrication of CuO/MoS_2_@gCN Nanocomposite for Effective Degradation of Methyl Orange
and Phenol Photocatalytically. Results Phys.

[75] Ren H.-R., Liu Y., Zhang Y.-T., Yan P.-J., Qin D.-D., Yao X.-Q. (2025). A Novel
Zn MOFs as a Multifunctional Sensitive Chemical Sensor for the Rapid
Detection of Nitroaromatic Compounds, Tetracycline, Fe^3+^, and Degradation for Methyl Orange. Inorg.
Chim. Acta.

[76] Wei L., Zhang X., Wang J., Yang J., Yang X. (2024). Synthesis
of Fe_2_O_3_/g-C_3_N_4_ Composite
with Efficient Photocatalytic Degradation for Methyl Orange. Inorg. Chem. Commun.

[77] Prasetyowati R., Harahap E. F. M., Saputri R. I., Swastika P. E., Fauzi F., Supardi, Warsono, Ariswan, Dwandaru W. S. B. (2024). Degradation of
Methyl Orange Dye Using Fe_3_O_4_/GO Photocatalyst
with Iron Derived from Coastal Glagah Kulon Progo Ore. Nano-Struct. Nano-Objects.

[78] Das A., Patra M., Kumar P. M., Bhagavathiachari M., Nair R. G. (2021). Role of type II heterojunction in ZnO–In2O3
nanodiscs for enhanced visible-light photocatalysis through the synergy
of effective charge carrier separation and charge transport. Mater. Chem. Phys.

[79] Ruan S., Huang W., Zhao M., Song H., Gao Z. (2020). A Z-Scheme
Mechanism of the Novel ZnO/CuO N-N Heterojunction for Photocatalytic
Degradation of Acid Orange 7. Mater. Sci. Semicond.
Process.

[80] Jia Y., Wang Z., Qiao X.-Q., Huang L., Gan S., Hou D., Zhao J., Sun C., Li D.-S. (2021). A Synergistic Effect
between S-Scheme Heterojunction and Noble-Metal Free Cocatalyst to
Promote the Hydrogen Evolution of ZnO/CdS/MoS_2_ Photocatalyst. Chem. Eng. J.

[81] Abebe B., Gupta N. K., Tsegaye D. (2024). A Critical Mini-Review on Doping
and Heterojunction Formation in ZnO-Based Catalysts. RSC Adv.

[82] Li Y., Wang X., Huo H., Li Z., Shi J. (2020). A Novel Binary
Visible-Light-Driven Photocatalyst Type-I CdIn_2_S_4_/g-C_3_N_4_ Heterojunctions Coupling with H_2_O_2_: Synthesis, Characterization, Photocatalytic
Activity for Reactive Blue 19 Degradation and Mechanism Analysis. Colloids Surf. A.

[83] Chen Y., Zhai B., Liang Y. (2019). Enhanced Degradation Performance
of Organic Dyes Removal by semiconductor/MOF/graphene Oxide Composites
under Visible Light Irradiation. Diamond Relat.
Mater.

[84] Chen Y., Zhai B., Liang Y., Li Y. (2020). Hybrid Photocatalysts
Using semiconductor/MOF/graphene Oxide for Superior Photodegradation
of Organic Pollutants under Visible Light. Mater.
Sci. Semicond. Process.

[85] Jabbar Z. H., Graimed B. H., Issa M. A., Ammar S. H., Ebrahim S. E., Khadim H. J., Okab A. A. (2023). Photocatalytic Degradation of Congo
Red Dye Using Magnetic Silica-Coated Ag_2_WO_4_/Ag_2_S as Type I Heterojunction Photocatalyst: Stability and Mechanisms
Studies. Mater. Sci. Semicond. Process.

[86] Qin H., Li W., Xia Y., He T. (2011). Photocatalytic Activity of Heterostructures
Based on ZnO and N-Doped ZnO. ACS Appl. Mater.
Interfaces.

